# Design, synthesis and antitumor evaluation of novel pyrazolo[3,4-*d*]pyrimidines incorporating different amino acid conjugates as potential DHFR inhibitors

**DOI:** 10.1080/14756366.2022.2142786

**Published:** 2022-11-16

**Authors:** Ibrahim M. Salem, Samia M. Mostafa, Ismail Salama, Osama I. El-Sabbagh, Wael A. H. Hegazy, Tarek S. Ibrahim

**Affiliations:** a Medicinal Chemistry Department, Faculty of Pharmacy, Suez Canal University, Ismailia, Egypt; b Medicinal Chemistry Department, Faculty of Pharmacy, Zagazig University, Zagazig, Egypt; c Department of Microbiology and Immunology, Faculty of Pharmacy, Zagazig University, Zagazig, Egypt; d Pharmacy Program, Department of Pharmaceutical Sciences, Oman College of Health Sciences, Muscat, Oman; e Department of Pharmaceutical Chemistry, Faculty of Pharmacy, King Abdulaziz University, Jeddah, Saudi Arabia; f Department of Pharmaceutical Organic Chemistry, Faculty of Pharmacy, Zagazig University, Zagazig, Egypt

**Keywords:** Pyrazolo[34-*d*]pyrimidine, *N*-acyl amino acids, methotrexate, DHFR inhibition, MCF-7 breast cancer cell line

## Abstract

The present study aimed to investigate the antitumor effect of simultaneous inhibition of dihydrofolate reductase (DHFR) enzyme. We designed some novel pyrazolo[3,4-*d*]pyrimidines bearing different amino acid conjugates as efficient antifolate agents attributable to their structural similarity with methotrexate (MTX) and MTX-related antifolates. All compounds were tested to screen their enzymatic inhibition against DHFR compared with the reference drug MTX and for their *in vitro* antitumor cytotoxicity against six MTX-resistant cancer cell lines. The flow cytometry indicated that the most potent compound **7f** arrested MCF-7 cells in the S-phase and induced apoptosis. Western blot for visualisation proved the ability of compound **7f** to induce the expression of proapoptotic caspases and Bax proteins in MCF-7 breast cancer cell line beside its ability to diminish the expression of antiapoptotic Bcl-2 protein. Molecular modelling studies concluded that compound **7f** displayed better binding energy than that of the normal ligand MTX.

**HIGHLIGHTS**
- New pyrazolo[3,4-*d*]pyrimidine derivatives **7a–m** which are structurally similar to the classical methotrexate (MTX) and MTX-related antifolates were synthesised as antitumor agents.
- Novel *N*-acyl amino acid compound **7f** exhibited marked DHFR inhibition activity that are parralel to both the molecular docking results and cytotoxic activity.
- Compound **7f** could induce the expression of proapoptotic caspases and Bax proteins in MCF-7 breast cancer cell line beside its ability to diminish the expression of antiapoptotic Bcl-2 protein.
- All prepared compounds obey Lipinski rule of five except compound **7f**.

## GRAPHICAL ABSTRACT

**Figure:**
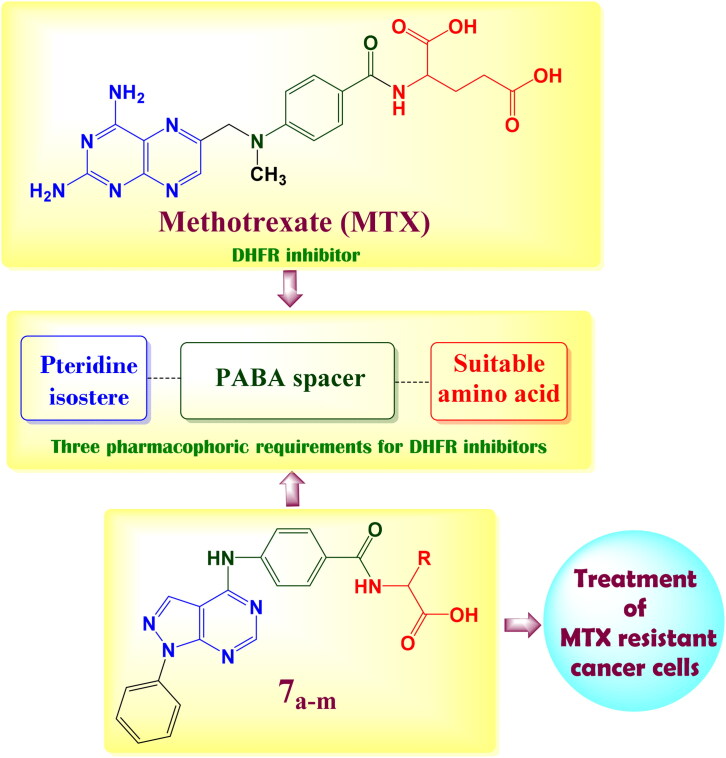

## Introduction

**Figure 1.**
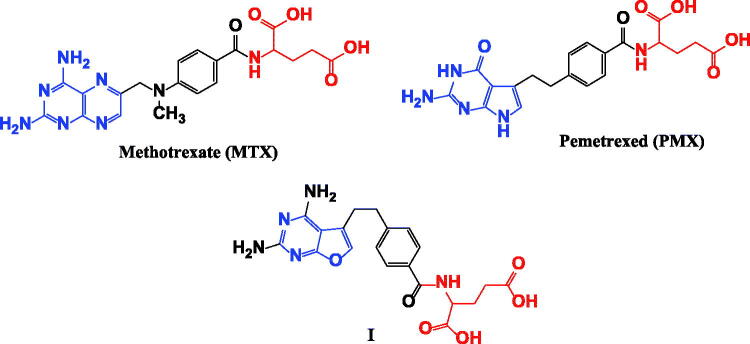
Structural requirements for DHFR inhibition activity.

Dihydrofolate reductase (DHFR) is a critical enzyme in folic acid metabolism; it promotes catalysis for conversion of dihydrofolate (DHF) to tetrahydrofolate (THF), which is essential for purine *de novo* synthesis and thymidylate synthesis in cell proliferation^1^, therefore inhibition of DHFR enzyme exhibits great antitumor activity as it suppresses *de novo* nucleotide biosynthesis leading to an imbalance of purine and pyrimidine precursors and rendering cells incapable of undergoing a proper DNA replication^2^. The most common dihydrofolate reductase inhibitors (DHFRIs) are the classical antifolates such as methotrexate (MTX), aminopterine, pralatrexate and pemetrexed (PMX)^3^. The chemical structure of MTX prototype is composed of 3 main important pharmacophores: pteridine nucleus, 4-amino benzoic acid and glutamic acid^4^ (Figure 1). With mismatch, pemetrexed (PMX) elucidates ascendant DHFR inhibition effect although it accommodates pyrrolo[2,3-*d*]pyrimidine nucleus instead of pteridine^5^ as well the furo[2,3-*d*]pyrimidine in analogue **I** was also reported as potential DHFR inhibitor^6^ as shown in Figure 1.

**Figure 2.**
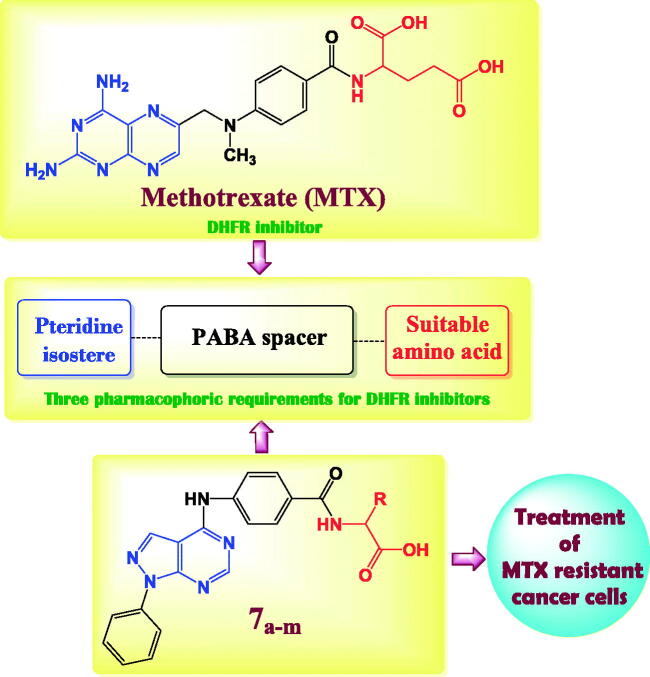
The structural approach between the synthesised novel pyrazolo[3,4-*d*]pyrimidines **7a–m** with MTX reference drug.

**Scheme 2.**
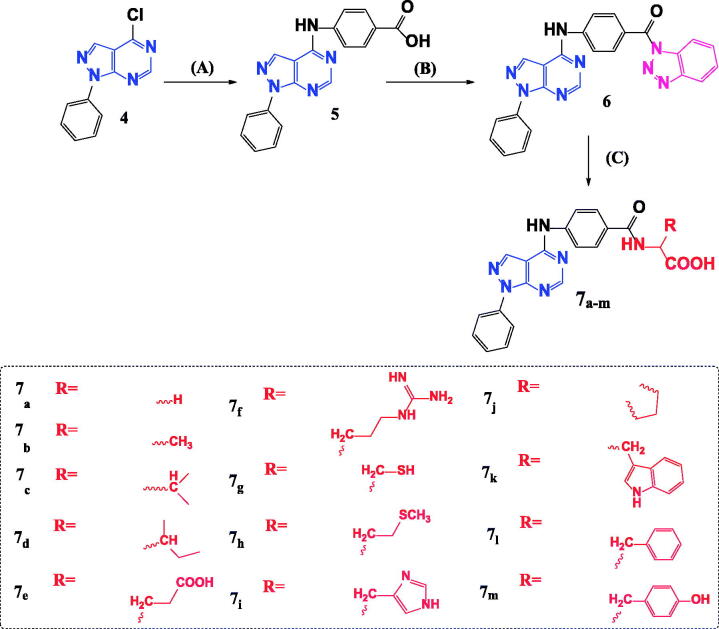
Synthetic pathway for preparation of novel *N-*acyl amino acid conjugates **7a–m**. Reagents and conditions: A = 4-aminobenzoic acid, isopropanol, heat, 16–18 h. B = 1,2,3 Benzotriazole, SOCI_2_, DCM, room temp. C = Amino acid, TEA, Acetonitrile, heat 70° C.

The previous findings encouraged us to synthesise novel pyrazolo[3,4-*d*]pyrimidine derivatives incorporate a different series of amino acid conjugates **7a–m** as shown in Scheme 2. Our aim is based on the isosteric replacement of the pteridine nucleus of MTX with pyrazolo[3,4-*d*]pyrimidine scaffold with the retention of 4-aminobenzoyl spacer and either glutamic acid or different series of amino acids to achieve a significant inhibitory level of DHFR enzyme as in Figure 2 and therefore, achieve considerable cytotoxic activities against different MTX-resistant cell lines. We selected different series of amino acid conjugates depending on their different hydrophilic or hydrophobic behaviours beside their different aliphatic or aromatic nature hoping to achieve superior DHFR inhibition activity that exceed classical antifolates and to treat the classical antifolates resistant cancer cell lines.

## Results and discussion

### Chemistry

**Scheme 1.**
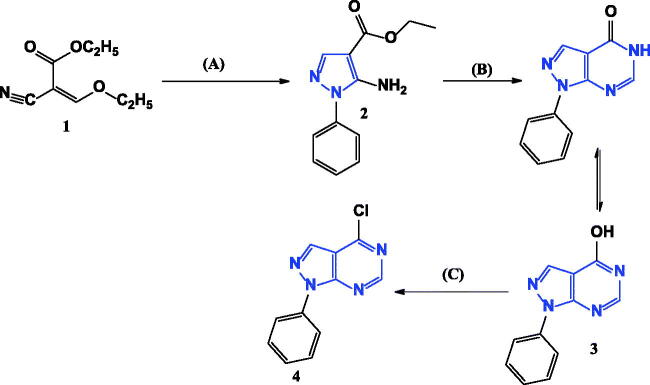
Synthesis of 4-chloro-1-phenyl-1*H*-pyrazolo[3,4-*d*]pyrimidine **4**. Reagents and conditions: A = Phenyl hydrazine, ethanol, 80° C, 4 h. B = HCONH_2_, 190° C, 8 h. C = POCI_3_, 106° C, 6 h.

Our starting synthetic material ethyl (ethoxymethylene)cyanoacetate **1** was cyclized with phenyl hydrazine to obtain ethyl 5-amino-1-phenyl-1*H*-pyrazole-4-carboxylate **2**^7^ which underwent further cyclisation with formamide to afford the pyrazolo[3,4-*d*]pyrimidinone compound **3**^8^ as shown in Scheme 1.

Compound **3** was chlorinated using phosphorous oxychloride to obtain 4-chloro-1-phenyl-1*H*-pyrazolo[3,4-*d*]pyrimidine **4**^9^ (Scheme 1) which reacted with 4-amino benzoic acid to give the key intermediate 4-{(1-phenyl-1*H*-pyrazolo[3,4-*d*]pyrimidin-4-yl)amino}benzoic acid (**5**)^10^ as shown in Scheme 2.

Reaction of our key intermediate **5** with 1*H*-benzotriazole afforded the novel *N*-acyl benzotriazole compound **6** that was condensed with a series of different amino acids to obtain *N*-acyl amino acids compounds (**7a–m**) (Scheme 2).

The novel *N*-acyl benzotriazole derivative **6** was characterised using melting point and TLC techniques in different solvent systems. The structure of compound **6** was confirmed using ^1^HNMR and ^13^CNMR whereas its ^1^HNMR revealed the absence of any signals in aliphatic region however; it comprised numerous signals in aromatic region integrated into 15 aromatic protons which are divided into; 2 doublet signals at 7.69 and 8.62 ppm integrated for 8 protons, 2 triplet signals at 7.39 and 7.59 ppm integrated for 5 protons. In addition, the appearance of two characteristic singlet signals at 8.23 ppm confirm the presence of 3-H and 6-H position of pyrazolo[3,4-*d*]pyrimidine nucleus. ^13^CNMR data of compound **6** elucidated 23 signals at aromatic region. Besides, the signal of carbonyl group carbon is at 161.09 ppm. Moreover, the chemical structures of 2-{4-[(1-phenyl-1*H*-pyrazolo[3,4-*d*]pyrimidin-4-yl)amino]benzamido}alkanoic acid (**7a–m**) were confirmed with their elemental analysis, ^1^HNMR and ^13^CNMR. ^1^HNMR spectra clarified that compounds **7a–h** and **7j** showed an apparent decrease in the number of aromatic signals to be integrated for 11 aromatic protons only; consequently, that explains the actual loss of 1*H*-benzotriazole as a good leaving group. In addition to, appearance of new signals in aliphatic region that vary from compound to the other. Compound **7a** showed singlet signal in aliphatic region at 3.91 ppm integrated to 2 protons of (CH_2_) of glycine, while compound **7b** illustrated 2 significant signals; one doublet signal at 1.43 ppm integrated to 3 protons of (CH_3_) group and the other quartette signal at the range of 4.40–4.46 ppm refer to 1 proton of (CH) at C_2_ of alanine amino acid. Furthermore, compound **7c** revealed 3 considerable signals in aliphatic region; at 0.96 ppm that represents a large doublet signal integrated to 6 protons of 2 (CH_3_) groups, a multiplet signal from 1.17 to 1.25 ppm integrated to 1 proton corresponding to (CH) at C_3_ of valine beside a doublet signal at 4.28 ppm indicates the proton of (CH) at C_2_. Beyond, compound **7d** showed doublet signal at 0.93 ppm corresponding to (CH_3_) group radiated from C_3_, a triplet signal at 1.31 ppm indicates the (CH_3_) group substituted at C_4_ of isoleucine, another multiplet signal at the range of 1.95–1.99 ppm performs (CH) at C_3_ beside a doublet signal at 4.38 ppm refer to (CH) at C_2_. Moreover, compound **7e** exhibited a quartette signal at the range of 1.98–2.11 ppm of aliphatic region due to (CH_2_) at C_3_ of glutamic acid alongside a triplet signal at 2.40 ppm integrated to 2 protons of (CH_2_) at C_4_ and a triplet peak at 4.43 ppm due to (CH) at C_2_. As well, compound **7f** revealed 3 signals indicate the 3 (CH_2_) groups of arginine amino acid, a multiplet signal appeared at 0.79–0.85 ppm corresponding to (CH_2_) at C_4_ besides, a quartette peak at 1.09–1.21 ppm due to (CH_2_) at C_3_, the triplet signal performs (CH_2_) at C_5_ of arginine become clear at 1.66 ppm. In addition to a triplet peak at 4.38 ppm corresponding to (CH) at C_2_ of arginine and an evident increase in the signals refer to NH groups due to presence of guanidine moiety. Too, compound **7g** demoed a doublet signal at 3.13 ppm integrated to 2 protons corresponding to (CH_2_) at C_3_ of cysteine along with a triplet signal at 4.69 ppm due to (CH) at C_2_. Also, compound **7h** displayed several peaks at aliphatic region; a quartette peak at 1.15–1.23 ppm corresponding to (CH_2_) at C_3_ of methionine, singlet signal at 2.08 ppm due to (–S–CH_3_) group and 2 triplet signals at 2.60 and 4.50 ppm represent the (CH_2_) at C_4_ and (CH) at C_2_ of methionine respectively. With a similar manner, compound **7j** elucidated 4 significant signals at aliphatic region due to pyrrolidine ring of proline. These aliphatic signals appeared at the range of 1.18–1.28 ppm and 2.25–2.30 ppm beside 3.40 ppm referring to (CH_2_) of pyrrolidine ring at C_4_, C_3_ and C_5_ respectively. Besides, a triplet peak at 4.42 ppm corresponding to C_2_ of pyrrolidine. In contrast, compound **7i** demonstrated an aromatic region with numerous signals integrated to 13 aromatic protons, this increase in the number of aromatic signals is owing to the presence of 2 singlet signals indicates (CH) at C_5_ and C_2_ of imidazole ring of histidine at 6.91 and 7.92 ppm respectively. Remarkably, compounds **7k–m** revealed another increase in the number of aromatic signals to be integrated to 16 aromatic protons in compounds **7k,l** and 15 aromatic protons in compound **7 m** that could be explained by removal of 1*H*-benzotriazole and increment of an aromatic amino acid such as tryptophan, phenyl alanine and tyrosine. Interestingly, all synthesised novel *N*-acyl amino acids **7a–m** show singlet signal represents the proton of (COOH) group at a range of 10.51–10.86 ppm except compound **7e** that demoed 2 singlet signals at 10.51 and 12.48 ppm that demonstrates the presence of 2 carboxylic groups. Furthermore, ^13^CNMR spectrum of compounds **7a–h** and **7j** clarified a perspicuous decrease in the number of aromatic carbons to 17 carbons rather than the 23 carbon signals presented at *N*-acyl benzotriazole compound **6** that proves the replacement of 1*H*-benzotriazole by an aliphatic amino acid. In addition, an appearance of new signals in aliphatic region such as the signals appeared at 18.19 and 50.57 ppm of compound **7b** representing C_3_ and C_2_ of alanine respectively. Moreover, compound **7e** exhibited 3 additional aliphatic signals at 26.95, 30.57 and 53.91 ppm corresponding to C_3_, C_4_ and C_2_ of glutamic acid respectively. In contrast, compounds **7k–m** showed a sudden increase in aromatic signals due to the addition of aromatic amino acid. Interestingly, the ^13^C NMR spectra of all prepared compounds demoed 1 signal corresponding to one carbonyl group except compound **7e** that exerts 2 signals at 175.47 and 176.74 ppm referring to 2 carbonyl groups that explain the presence of glutamic acid.

### Biological evaluation

#### Cytotoxic activity of synthesised compounds

The SRB colorimetric assay was employed to assess the synthesized compounds’ cytotoxic effects on several cancer cell lines (Table 1)^11–14^. The synthesized compounds especially **7c, 7d, 7f, 7i, 7j** and **7l** inhibited the cellular proliferation of cancer cell lines at lower IC_50_ values. Compounds **7e, 7g** and **7m** inhibited the proliferation of cancer cells at higher IC_50_ in compression to above compounds. The highest IC_50_ to almost cancer cell lines were observed for compounds **6, 7a, 7h, 7k** and **7b**. Noticeably, the lowest IC_50_ values were observed on MCF-7 breast cancer line when treated with the tested compounds, especially compounds **7c, 7d, 7f, 7i, 7j** and **7l**. Furthermore, the effect of tested compounds was evaluated on the normal immortalized HPDE cell line. All synthesized compounds have no toxicity to the normal cells with higher IC_50_ exceeding 5.61 μM (Table 1).

**Table 1.** IC_50_ values of compound **6** and novel *N*-acyl amino acid conjugates **7a–m** in different cell lines.

Compounds	IC_50_ (µM)							
	HeLa	PC-3	HCT-116	BxPC-3	HepG-2	MCF-7	DHFR	HPDE
**6**	15.11 ± 1.21	14.91 ± 1.24	17.26 ± 2.18	14.21 ± 1.36	12.98 ± 1.86	11.91 ± 1.10	7.99 ± 0.16	>50
**7a**	19.45 ± 1.21	23.03 ± 1.03	28.24 ± 1.93	19.45 ± 1.56	15.45 ± 0.96	15.29 ± 1.29	13.27 ± 0.24	>50
**7b**	29.21 ± 1.90	34.24 ± 2.61	29.56 ± 1.91	31.23 ± 2.80	19.23 ± 1.87	35.67 ± 2.09	42.49 ± 0.78	>50
**7c**	9.39 ± 0.25	11.25 ± 0.34	10.60 ± 1.10	10.25 ± 1.01	12.25 ± 1.14	6.56 ± 0.18	0.61 ± 0.01	>50
**7d**	8.09 ± 0.51	9.99 ± 0.81	10.09 ± 1.01	11.19 ± 0.41	9.09 ± 0.71	5.51 ± 0.28	0.43 ± 0.01	>50
**7e**	13.11 ± 1.01	13.81 ± 1.14	15.26 ± 1.18	15.21 ± 1.15	15.98 ± 0.80	11.91 ± 1.10	1.83 ± 0.04	>50
**7f**	6.99 ± 1.11	6.29 ± 0.56	5.48 ± 0.04	7.09 ± 0.09	5.12 ± 0.01	4.65 ± 0.17	0.31 ± 0.01	>50
**7g**	12.29 ± 1.20	13.21 ± 1.52	12.21 ± 1.20	15.21 ± 0.22	10.21 ± 0.92	10.78 ± 1.10	1.28 ± 0.03	>50
**7h**	18.01 ± 1.20	17.01 ± 2.02	19.34 ± 0.85	17.87 ± 1.03	16.99 ± 0.92	19.98 ± 2.01	8.47 ± 0.18	>50
**7i**	10.91 ± 1.99	12.78 ± 2.01	13.04 ± 1.01	10.54 ± 3.01	9.26 ± 1.56	13.21 ± 1.21	0.82 ± 0.02	>50
**7J**	9.29 ± 0.20	11.17 ± 0.70	8.10 ± 0.20	6.19 ± 0.43	10.29 ± 1.20	8.19 ± 1.20	0.54 ± 0.01	>50
**7k**	19.53 ± 1.10	19.88 ± 1.35	18.02 ± 1.23	19.08 ± 1.23	25.54 ± 0.14	28.90 ± 1.91	7.00 ± 0.17	>50
**7l**	9.05 ± 1.20	10.08 ± 0.90	12.99 ± 0.89	11.45 ± 0.76	13.19 ± 1.29	10.67 ± 1.09	0.66 ± 0.01	>50
**7m**	11.81 ± 1.13	12.72 ± 1.87	13.32 ± 2.21	11.99 ± 1.14	14.25 ± 0.98	11.30 ± 1.28	1.34 ± 0.03	>50
**Doxorubicin**	5.57 ± 0.51	9.01 ± 0.09	5.52 ± 0.13	7.23 ± 0.10	4.6 ± 0.44	4.25 ± 0.15	–	>50
**Methotrexate**	294.77 ± 0.51	42.33 ± 7.25	99.98 ± 0.22	29.11 ± 0.31	800.24 ± 0.16	66.11 ± 0.15	5.61 ± 0.11	>50

#### Inhibitory effect of tested compounds on human DHFR enzyme

**Figure 3.**
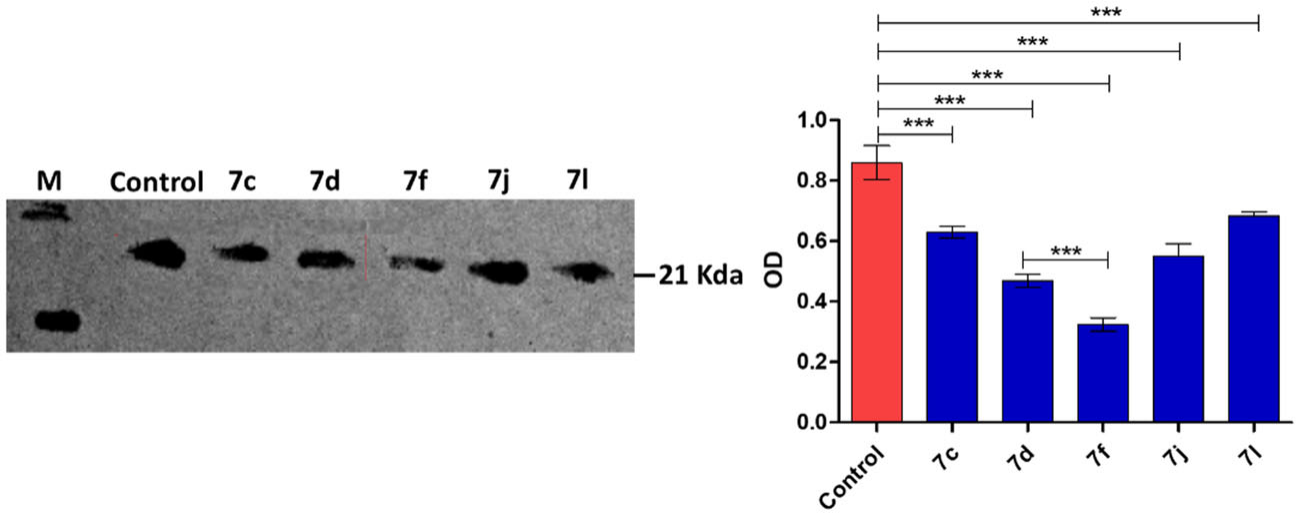
Western blot quantification anti-DHFR effects of compounds **7c, 7d, 7f, 7j** and **7l** in MCF-7 breast cancer cell.

Compounds **7c, 7d, 7f, 7i, 7j** and **7l** inhibited DHFR at considerable low IC_50_ < 1 µM in compression to MTX the reference drug (IC_50_ = 5.61 µM) (Table 1). Then compounds **7e, 7g** and **7m** inhibited DHFR at IC_50_ range 1–5 µM which is lower than observed with the MTX. The highest DHFR (IC_50_) was observed for all compounds **6, 7a, 7h, 7k** and in particular **7b**. Stimulatingly, the DHFR inhibition by compounds **7c, 7d, 7f, 7j** and **7l** was in a great compliance with their cytotoxic effects on MCF-7 cancer cell line^15^. The efficient DHFR inhibition with considerable cytotoxic effects on cancer cell lines could elect the compound in order **7c, 7d, 7f, 7j** and **7l** to be possible anticancer candidates. For more investigation, the inhibitory effects of compounds **7c, 7d, 7f, 7j** and **7l** on the expression of DHFR in the MCF-7 cancer cell line were further western blot quantified (Figure 3). Obviously, the nominated compound especially the compound **7f** significantly diminished the DHFR expression that attests their anti-DHFR activities.

**Figure 4.**
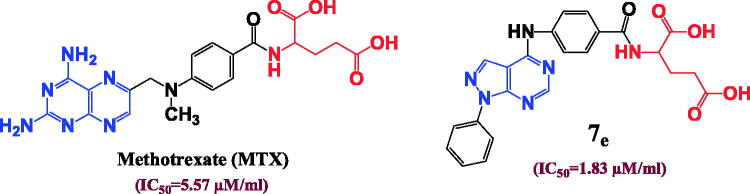
Isosteric approach of compound **7e** and MTX along with DHFR inhibition IC_50_ (µM) values.

The structure activity relationship clarified that the isosteric replacement of pteridine ring in MTX by pyrazolo[3,4-*d*]pyrimidine nucleus improve the DHFR inhibition activity in the majority of the prepared compounds. Consequently, compound **7e** is considered the direct isostere of MTX with retention of 4-aminobenzoyl and glutamic acid moieties exhibits superior DHFR inhibition activity (IC_50_ = 1.83 µM) comparable to MTX (IC_50_ = 5.57 µM) (Figure 4).

**Figure 5.**
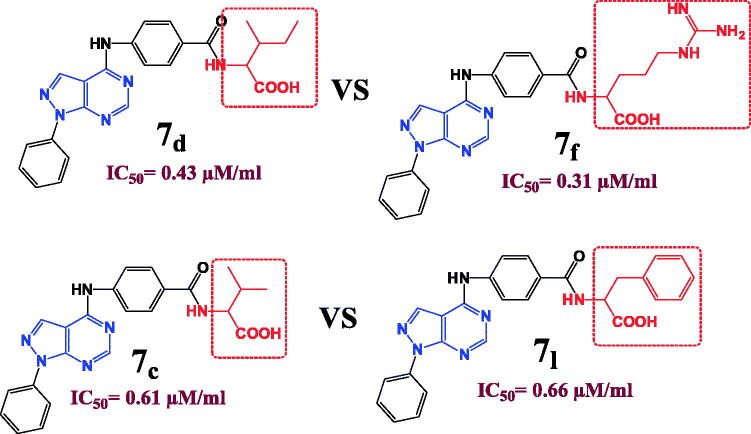
The effect of the structural differences between compounds **7c, 7d, 7f** and **7l** on their IC_50_ (µM) values.

In a strangest manner, hydrophilic and hydrophobic nature of the conjugated amino acid is insignificant parameter as both compounds **7d** with hydrophobic (Ile) amino acid and **7f** with hydrophilic (Arg) amino acid elucidated near considerable DHFR inhibition activities (IC_50_= 0.43 and 0.31 µM), respectively and near cytotoxic activities against different cell lines. In addition, the aliphatic and aromatic nature of the inserted amino acid also has no remarkable effect on the DHFR inhibition activities since both compounds **7c** and **7l** have approximate IC_50_ values of 0.61 and 0.66 µM, respectively although compound **7c** possess (Val) aliphatic amino acid while compound **7l** enclose (Phe) aromatic one and with also approximate cytotoxic activities as in (Figure 5). In consequence, the solubility parameter beside the aliphatic and aromatic nature of the inserted amino acid is not important for DHFR inhibition activity in compounds bearing pyrazolo[3,4-*d*]pyrimidine ring instead of pteridine. However, the isosteric replacement of pteridine ring by pyrazolo[3,4-*d*]pyrimidine revealed a considerable increase in the DHFR inhibition activity.

#### Apoptosis induction in cancer cells

Induction of cancer cells apoptosis play the crucial role in diminishing their proliferation and metastasis, and so the anti-apoptotic effect indicates a possible anticancer activity^16^^,^^17^. Apoptotic process involves diverse metabolic reactions that can be activated or inhibited. It is well documented that suppression of cancer antiapoptotic proteins as well as activation proapoptotic proteins indicates efficient antitumor activity^11–14^. In this direction, the most efficient anti-DHFR compound with the least cytotoxicity on MCF-7 cells "compound **7f**” was selected for further anti-apoptotic activity evaluation.

#### Compound 7f induces the expression of proapoptotic proteins in MCF-7 breast cancer cell line

**Figure 6.**
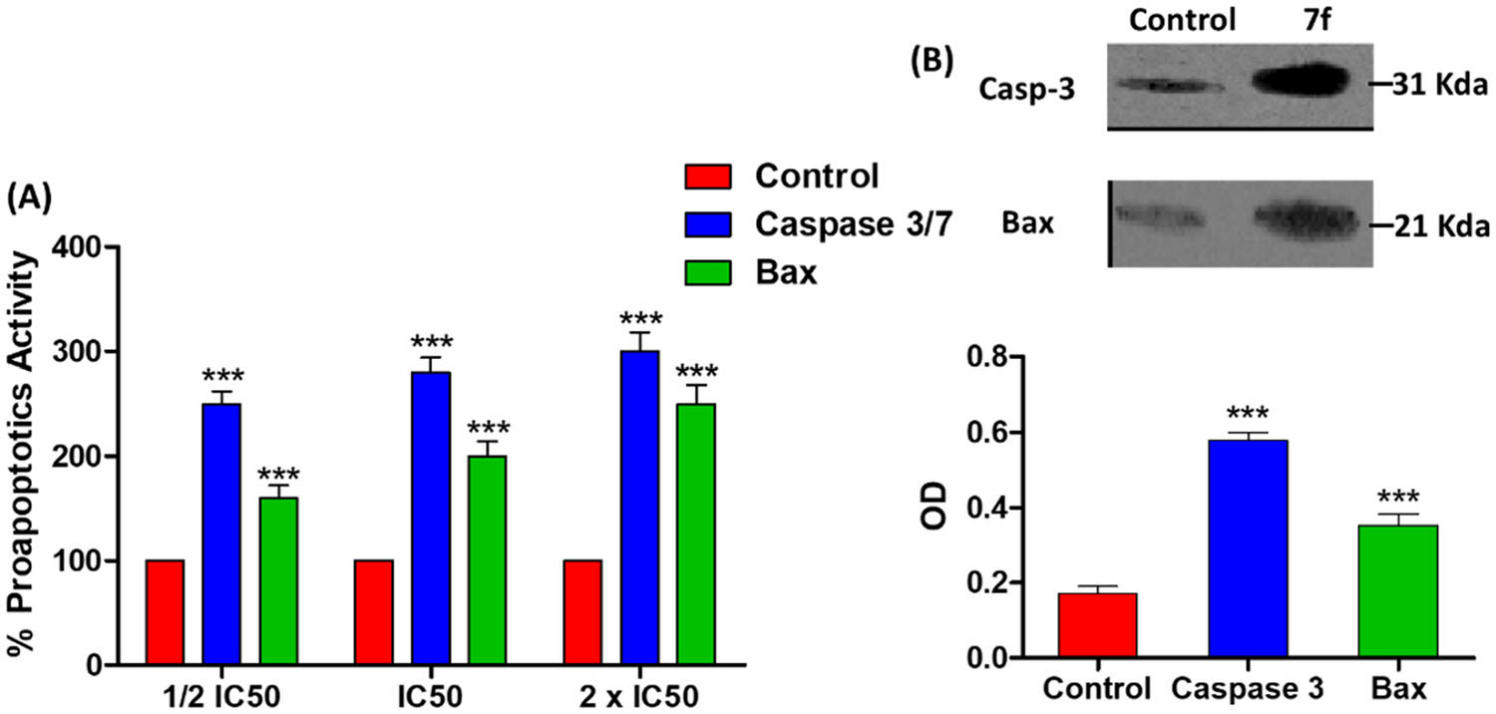
The compound **7f** increases the levels of caspases in breast cancer cell line MCF-7. (A) Coulometric assay of caspase 3/7 and Bax levels, the data are presented as percentage change from untreated control. (B) Western blot gel of caspase-3 and Bax and quantification of the intensities of the bands in western blot.

Caspases as well as Bax are proapoptotic proteins that are regularly triggered during apoptosis, and up-regulation of their expression in cancer cells indicates antitumor activity^11^^,^^14^. In this context, the effect of **7f** at IC_50_ on the levels of caspase 3/7 and Bax proteins was evaluated. In dose dependent manner, the compound **7f** significantly surge the release of caspase 3/7 and Bax proteins (Figure 6A). For further testing the colorimetric assays of caspases or Bax proteins, a western blot was performed to visualise and quantify their expressions. Compound **7f** significantly increased the expression of both caspase 3 and Bax proteins (Figure 6B). The β-actin (42 KDa) was used as internal reference to validate the western blots. The levels of the expressed β-actin remain unaffected in the presence of different tested compounds (Supporting Information).

#### Compound 7f diminishes the expression of antiapoptotic Bcl2 protein

**Figure 7.**
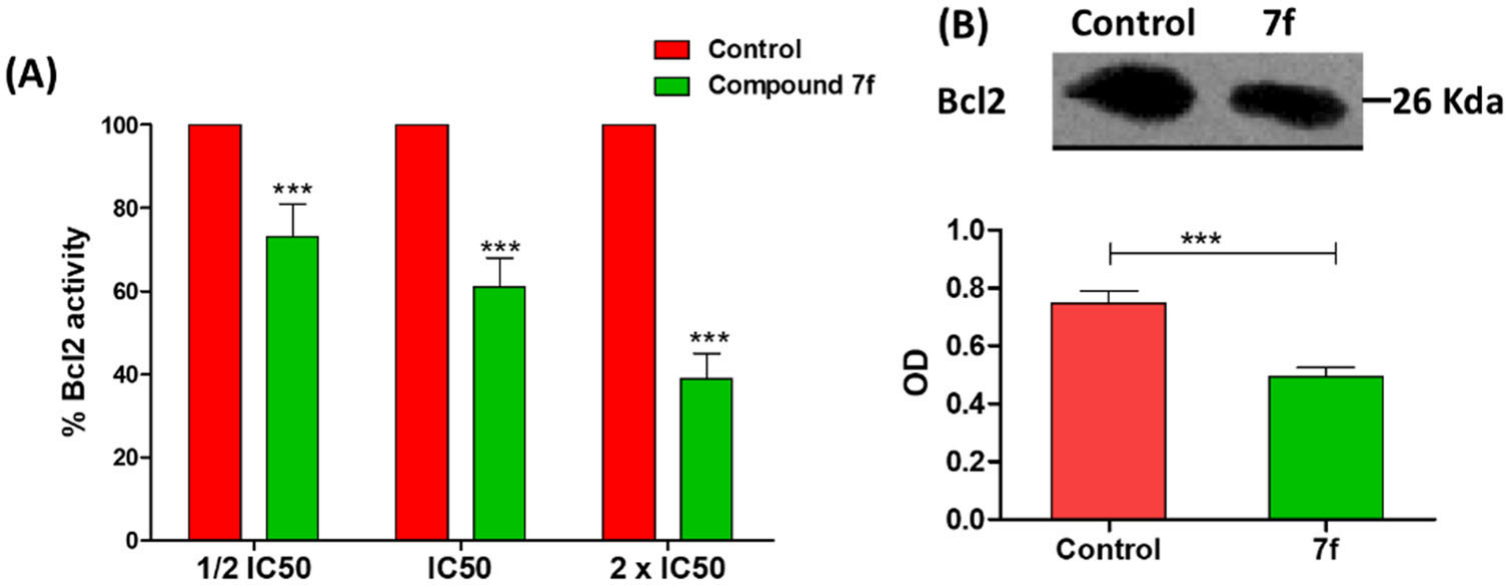
(A) The compound **7f** decreases the level of Bacl2 in breast cancer cell line MCF-7. (A) ELISA assay of Bcl2 level, the data are presented as percentage change from untreated control. (B) Western blot gel of Bcl2 and quantification of the intensities of the bands in western blot.

B-cell lymphoma-2 (Bcl-2) protein regulates the apoptosis; it is an antiapoptotic protein to facilitate the cancer cells survival under stressful circumstances via averting the proapoptotic signals^11^^,^^14^. To attest the compound **7f** apoptotic effect, Bcl-2 level was quantified in MCF-7 breast cancer cell line. Compound **7f** significantly reduced the Bcl-2 production in a dose-dependent manner (Figure 7A). Furthermore, the effect of compound **7f** at IC_50_ on the expression of Bcl-2 was visualised and quantified using western blot. Compound **7f** significantly down-regulated the expression of Bcl-2 (Figure 7B). The β-actin (42 KDa) was used as internal reference to validate the western blots. The levels of the expressed β-actin remain unaffected in the presence of different tested compounds (Supporting Information).

#### Flow cytometric cell cycle analysis

**Figure 8.**
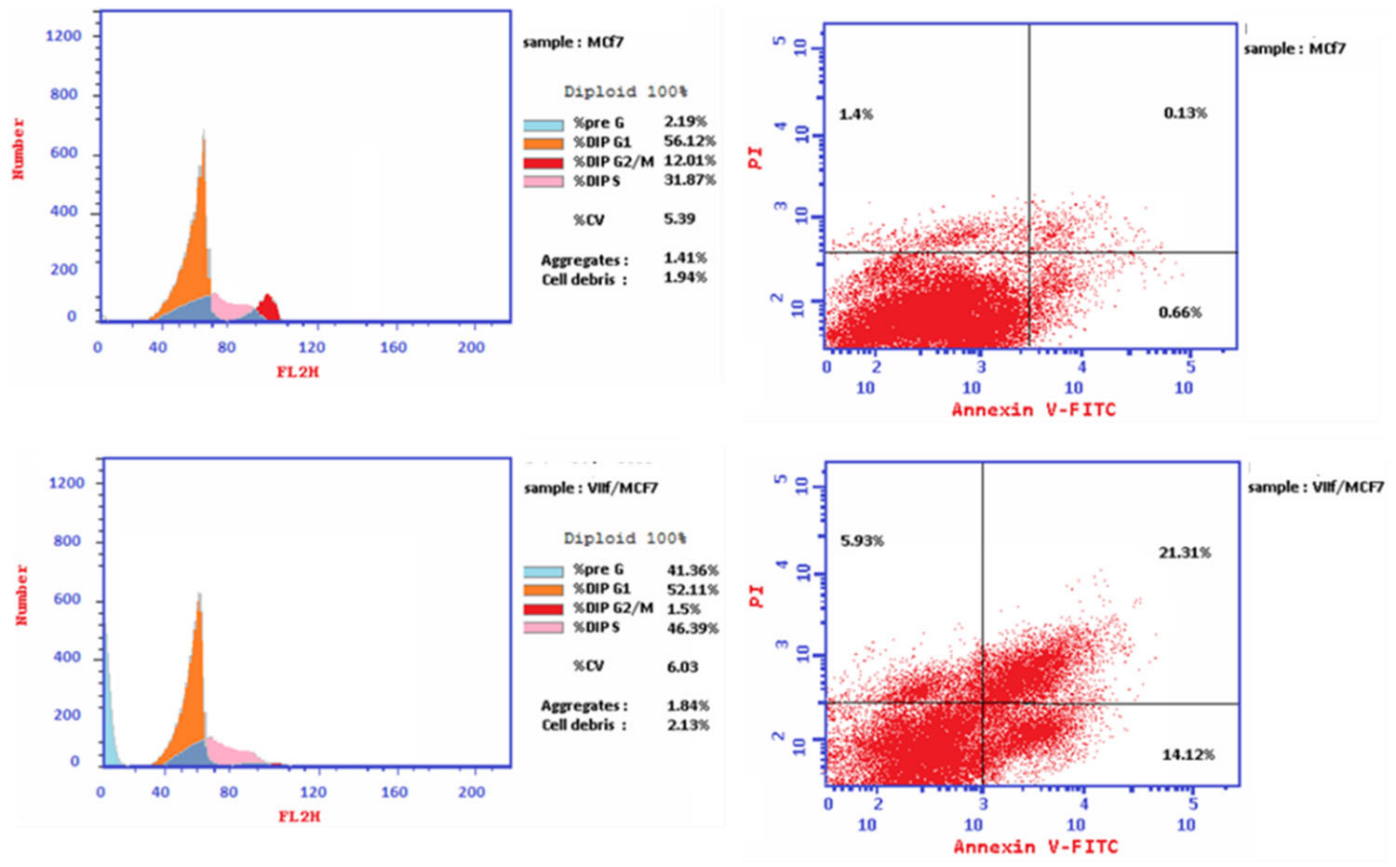
Flow cytometric cell cycle analysis upon treatment of MCF-7 breast cancer cell line with the compound **7f**.

Cell cycle analysis was done for the compound **7f** against MCF-7 breast cancer cell line^11^^,^^14^. Clearly, compound **7f** enhanced the accumulation proportion of cells at the pre-G1 phase from 2.19 to 41.36%, and the accumulation percentages in S phase from 31.87 to 46.36%. The decrease in the accumulation percentage of cells at G2/M phase from 12.01 to 1.5% after treatment with the compound indicated that the compound **7f** arrested cell cycle at S phase (Table 2). Moreover, the percentage of cell apoptosis was markedly increased from 0.13% for control MCF-7 cells to 21.31% in treated cells (Table 3; Figure 8). The proportion of late apoptosis is greater than that of early, which evidences that compound **7f** irreversibly induced apoptosis.

**Table 2.** Cell cycle analysis and apoptosis detection of the compound **7f**.

Compound	%G0-G1	%S	%G2/M	%Pre-G1	Comment
**7f/MCF7**	52.11	46.39	1.5	41.36	Cell growth arrest at S
**Cont.MCF7**	56.12	31.87	12.01	2.19	

**Table 3.** Apoptotic assay of the compound **7f**.

Compound	Apoptosis			Necrosis
	Total	Early	Late	
**7f/MCF7**	41.36	14.12	21.31	5.93
**Cont.MCF7**	2.19	0.66	0.13	1.4

### Molecular modeling

**Figure 9.**
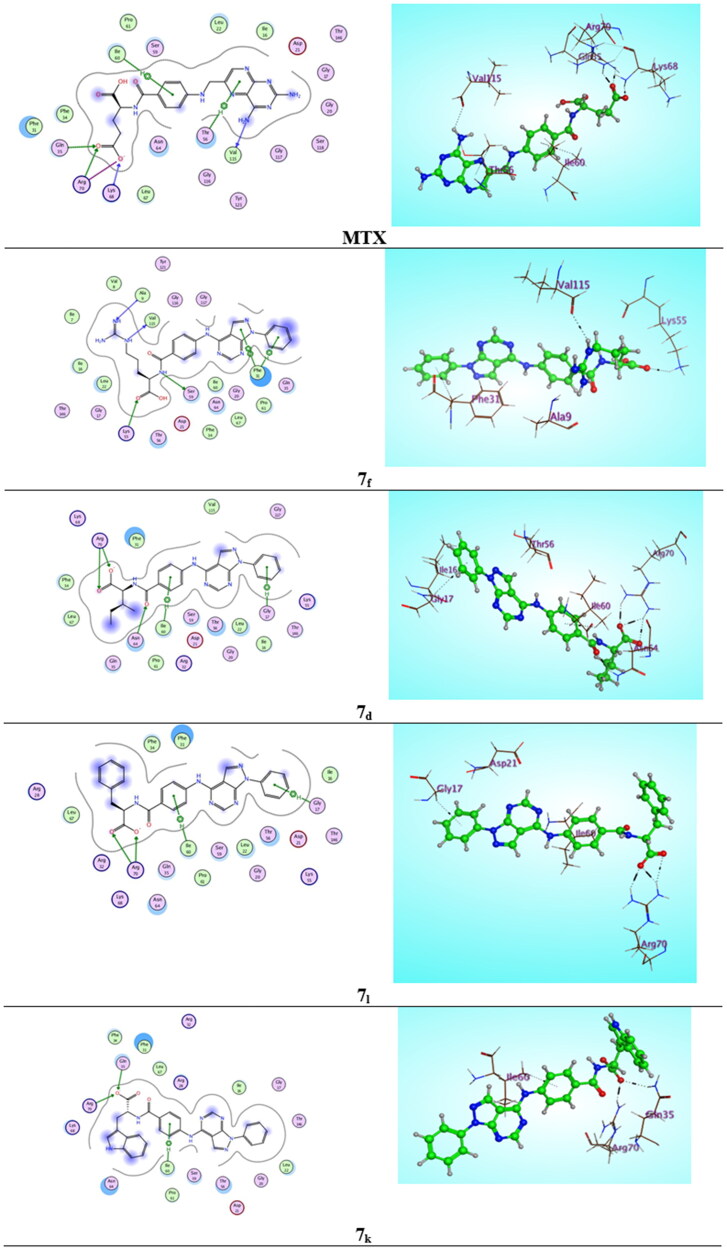
Two-dimensional (2 D) and three-dimensional (3 D) binding modes and residues involved in the energy minimised docking pose of the novel compounds **7f, 7d, 7l, 7k** and **MTX** against DHFR (pdb ID: 1U72).

The original ligand methotrexate (MTX) was extracted and docked into the active site of DHFR enzyme (pdb ID: 1U72)^18^. Validation of docking algorithm was achieved by re-docking MTX into the active site of DHFR, which was found to retrieve the reported X-ray crystal structure binding mode of MTX, with root mean square difference (RMSD) between the top docking pose and original crystallographic geometry of 1.263 A°. A stick representation of side-chain residues Ile60, Leu22, Arg70, Val115, Phe31, Phe34 and Gln35 of DHFR (pdb code ID: 1U72) in contact with MTX normal ligand as showed in (Figure 9). The crucial binding interactions attributable to MTX were; an important (HBD) effect due to 4-amino group of pteridine moiety with Val115 residue, besides the arene-H interaction of pteridine ring with Thr56 residue. In addition, there is a significant salt bridge caused by the γ-glutamate moiety of MTX with Arg70 accompanied with the (HBA) effect of both carbonyl and anionic carboxylate oxygens with Gln35 and Lys68 respectively. Unfortunately, previous studies of hDHFR have shown that certain mutations at Leu22, Phe31, Phe34, Gln35 and Arg70 residues could yield catalytically-active MTX-resistant mutants^19^. From this point, we investigated pyrazolo[3,4-*d*]pyrimidine derivatives **6** and **7a–m** and subsequently flexibly docked them into the binding site of DHFR compared with MTX as mentioned in Table 4.

**Table 4.** Docking scores (kcal/mol) and calculated parameters of Lipinski rule of prepared compounds **6, 7a–m** and methotrexate (MTX).

Compound	S	MW	Log P	nHBD	nHBA	druglike
**6**	−7.9944	432.4470	3.6975	1	9	1
**7a**	−7.7926	387.3790	1.5905	3	9	1
**7b**	−8.1547	401.4060	2.1735	2	9	1
**7c**	−8.2306	429.4600	3.1455	2	9	1
**7d**	−8.6795	443.4870	3.5875	2	9	1
**7e**	−8.5288	458.4340	1.9885	2	11	1
**7f**	−9.2584	487.5240	1.9135	7	12	0
**7g**	−8.0664	433.4720	2.2835	2	9	1
**7h**	−8.2034	461.5260	2.8285	2	9	1
**7i**	−8.5619	467.4690	1.4045	3	11	1
**7J**	−8.0525	428.4520	2.5615	2	9	1
**7k**	−9.0760	516.5410	4.0745	3	10	1
**7l**	−8.7263	477.5040	3.7085	2	9	1
**7m**	−9.0747	493.5030	3.4005	3	10	1
**MTX**	−8.3951	439.4120	−0.5874	7	13	0

Parallel to the *in vitro* DHFR enzyme assay screening (IC_50_) results, compound **7f** energy minimised pose elucidated binding energy of (S = −9.2584 kcal/mol) that is better than that of the normal ligand MTX (S = −8.3951 kcal/mol) due to presence of ᴨ-ᴨ* stacking interaction between pyrazole ring of pyrazolo[3,4-*d*]pyrimidine nucleus and Phe31 residue and arene-H interaction between 1-phenyl ring with the same Phe31 amino acid with bond lengths of 3.10 and 3.25 A° respectively, as well 2 (HBD) interactions were demonstrated by 2 NH groups of guanidine moiety with Ala9 residue and the key Val115 residue separately with bond energies of −0.8 and −1.4 Kcal/mol successively. Moreover, the docking results of the most stable conformer of compound **7d** revealed scoring function of (−8.6795 Kcal/mol) that also exceed MTX as a result of considerable salt bridge between carboxylate group and the crucial amino acid Arg70 (2.49 A°) with binding energy from (−1.9 to −7.6 Kcal/mol), also there were 2 arene-H interactions between 2 phenyl rings with Gly17 and Ile60 beside, an important (HBA) interaction of carbonyl group with Asn64 (2.04 A°) through bonding energy of −1.8 Kcal/mol. Interestingly, the energy minimised conformer of compound **7 l** established 2 binding interactions (S = − 8.7263 Kcal/mol) one of them as arene-H bond between 1-phenyl ring and Gly17 with bond length of (3.23 A°) and the other as the imperative salt bridge of the carboxylate moiety with the key Arg70 residue (bond energy from −0.8 to −7.6 kcal/mol). In atypical manner, compound **7k** showed docking scoring function of (−9.0760 kcal/mol) that is better than that of MTX normal ligand (−8.3951 kcal/mol) due to the dual interaction effect of the carboxylate moiety with Arg70 residue by salt bridge and with Gln35 by (HBA) effect (−1.8 Kcal/mol), beside the arene-H interaction arise between phenyl ring and Ile60 (−0.7 Kcal/mol), however it exhibited inferior *in vitro* IC_50_ value of 7 µM (MTX = 5.57 µM) as presented in Figure 9.

In addition, the parameters of Lipinski rule were calculated for all prepared novel compounds **6, 7a–m**. Profitability, all prepared compounds obey Lipinski rule of five except compounds **7f** due to presence of guanidine moiety of arginine amino acid that increase the number of (HBD) groups into 7 and the number of (HBA) groups into 12 as predicted in Table 4.

## Conclusions

It can be concluded that new pyrazolo[3,4-*d*]pyrimidines bearing different amino acid conjugates (**6, 7a–m**) were prepared as efficient antifolate agents due to their structural similarities with methotrexate (MTX). It was found that pyrazolo[3,4-*d*]pyrimidines **7c, 7d, 7f, 7i, 7j** and **7l** inhibited DHFR at considerable lower IC_50_ values than MTX reference drug beside their significant cytotoxic effects on many MTX-resistant cancer cell lines. Pyrazolo[3,4-*d*]pyrimidine **7f** bearing arginine amino acid is the most active antitumor agent against MCF-7 breast cancer cell line, DHFR inhibitor, as well it could arrest MCF-7 cells in the S-phase and induce apoptosis. Compound **7f** was proven to induce the expression of proapoptotic caspases and Bax proteins in MCF-7 breast cancer cell line beside its ability to diminish the expression of antiapoptotic Bcl-2 protein. Molecular modelling studies revealed that compound **7f** displayed binding energy of (S = −9.2584 kcal/mol) that is better than that of the normal ligand MTX (S = −8.3951 kcal/mol). Finally, compound **7f** require further future investigation as antitumor drug against MTX-resistant cancers because of its potential DHFR inhibitory activity.

## Experimental

### Chemistry

All starting materials were purchased from Sigma-Aldrich (Germany). Melting points (°C) were determined with a Gallenkamp melting point apparatus (London, UK), and are uncorrected. ^1^HNMR and ^13^CNMR were performed in NMR department, Faculty of Science, Mansoura University, Mansoura City, Egypt. ^1^HNMR spectra were recorded on Varian Mercury-500 (500 MHz) (Palo Alto, CA, USA), Varian Gemini-300BB (300 MHz) (Foster City, CA, USA), and Bruker 400 MHz AV III (400 MHz) (Biocity’s, CA, USA) using dimethyl sulfoxide (DMSO)-d_6_ as a solvent and tetramethylsilane (*TMS*) as internal standard (chemical shift in *δ*, ppm). ^13^CNMR spectra were recorded on Varian Gemini-300BB (100 MHz) (Foster City, CA, USA). All reactions were monitored by thin-layer chromatography (TLC) using Silica gel 60 GF254 (E-Merck-Germany) and were visualised by iodine vapours or by UV-lamp at wavelength *λ* 254 nm.

#### General procedure for synthesis of {1H-benzo[d][1,2,3]triazol-1-yl}{4-[(1-phenyl-1H-pyrazolo-[3,4-d]pyrimidin-4-yl)-amino]phenyl}-methanone (6)

To (1.4 g, 12 mmol) benzotriazole dissolved in (50 ml) CH_2_Cl_2_, (0.26 ml) SOCl_2_ (3.6 mmol) were added dropwise. The mixture was stirred at room temperature for 30 min, followed by the addition of the carboxylic acid containing compound **5** (1 g, 3 mmol) and the reaction was allowed to stir for an additional 2 h at room temperature. The reaction mixture was dried, then dissolved in ethyl acetate followed by washing using dilute saturated Na_2_CO_3_ solution. The organic layer was dried over anhydrous sodium sulphate. Hexane (50 ml) was added to the filtrate, and then the solid obtained was dried under vacuum to give the *N*-acyl benzotriazole **6.**

Yield: 92%. m.p. 307–309 °C; ^1^H NMR (500 MHz, DMSO-d_6_) δ: 4.00 (s, 1H, NH), 7.39 (t, *J* = 8.0 Hz, 2H, Ar-H), 7.59 (t, *J* = 8.0 Hz, 3H, Ar-H), 7.69 (d, *J* = 12.0 Hz, 5H, Ar-H), 8.23 (s, 2H, Ar-H), 8.62 (d, *J* = 12.0 Hz, 3H, Ar-H) ppm; ^13^CNMR (100 MHz, DMSO-d_6_) δ: 100.31(Ar-C), 113.85(Ar-C), 118.18(2 Ar-C), 119.87(Ar-C), 121.26(2 Ar-C), 124.76(Ar-C), 126.57(Ar-C), 127.60(2 Ar-C), 128.39(Ar-C), 129.36(3 Ar-C), 136.28(Ar-C), 137.49(Ar-C), 139.42(Ar-C), 143.01(Ar-C), 144.73(Ar-C), 152.58(Ar-C), 152.89(Ar-C), 154.70(Ar-C), 161.09(C = O) ppm. Anal. Calcd. for C_24_H_16_N_8_O: C, 66.66; H, 3.73; N, 25.91 Found: C, 66.74; H, 3.65; N, 26.07.

#### General procedure for synthesis of 2-{4-[(1-Phenyl-1H-pyrazolo[3,4-d]pyrimidin-4-yl)amino]-benzamido}-alkanoic acid (7a–m)

Suitable amino acid (0.55 mmol) was dissolved in H_2_O (2 ml) using 0.13 ml triethyl amine (0.9 mmol), then it is added portionwise to the solution of synthesised novel *N*-acyl benzotriazole **6** (0.2 g, 0.5 mmol) in CH_3_CN (15 ml). The mixture was heated at 70 °C for 4–6 h until complete consumption of compound **6** as monitored by TLC. The solvent was evaporated under reduced pressure. The residue was then washed by 4 N HCl (10 ml), water (10 ml) and filtered then dried to give the purposed compounds **7a–m**.

#### 2-{4-[(1-Phenyl-1H-pyrazolo[3,4-d]pyrimidin-4-yl)amino]benzamido}acetic acid (7a)

Yield: 82%. m.p. 302–304 °C; ^1^H NMR (500 MHz, DMSO-d_6_) δ: 1.92 (s, 1H, NH), 3.91 (s, 2H, CH_2_), 4.01 (s, 1H, NH), 7.38 (t, *J* = 8.0 Hz, 1H, Ar-H), 7.58 (t, *J* = 8.0 Hz, 2H, Ar-H), 8.03 (d, *J* = 12.0 Hz, 4H, Ar-H), 8.22 (s, 2H, Ar-H), 8.65 (d, *J* = 20.0 Hz, 2H, Ar-H), 10.57 (s, 1H, COOH) ppm; ^13^C NMR (100 MHz, DMSO-d_6_) *δ*: 43.27 (CH_2_), 100.31(Ar-C),120.48 (2 Ar-C), 121.26 (2 Ar-C), 126.57 (Ar-C), 127.43 (2 Ar-C), 128.70 (Ar-C), 129.33 (2 Ar-C), 136.28 (Ar-C), 139.42 (Ar-C), 144.43 (Ar-C), 152.58 (Ar-C), 152.89 (Ar-C), 154.70 (Ar-C), 169.25 (C = O), 172.44 (COOH). Anal. Calcd. for C_20_H_16_N_6_O_3_: C, 61.85; H, 4.15; N, 21.64 Found: C, 61.97; H, 4.22; N, 21.80.

#### 2-{4-[(1-Phenyl-1H-pyrazolo[3,4-d]pyrimidin-4-yl)amino]benzamido}propanoic acid (7b)

Yield: 89%. m.p. 311–313 °C; ^1^H NMR (500 MHz, DMSO-d_6_) *δ*: 1.43 (d, *J* = 4.0 Hz, 3H, CH_3_ C_3_), 1.94 (s, 1H, NH), 3.99 (s, 1H, NH), 4.40–4.46 (q, *J* = 8.0 Hz, 1H, CH C_2_), 7.38 (t, *J* = 8.0 Hz, 1H, Ar-H), 7.57 (t, *J* = 8.0 Hz, 2H, Ar-H), 8.02 (d, *J* = 8.0 Hz, 4H, Ar-H), 8.21 (s, 2H, Ar-H), 8.63 (d, *J* = 8.0 Hz, 2H, Ar-H), 10.51 (s, 1H, COOH) ppm; ^13^C NMR (100 MHz, DMSO-d_6_) *δ*: 18.19 (CH_3_, C_3_), 50.57 (CH, C_2_), 100.33 (Ar-C),119.92 (2 Ar-C),121.25 (2 Ar-C), 126.52 (Ar-C), 127.45 (2 Ar-C), 128.39 (Ar-C), 129.37 (2 Ar-C), 136.26 (Ar-C), 139.40 (Ar-C), 144.89 (Ar-C), 152.56 (Ar-C), 152.87 (Ar-C), 154.71 (Ar-C), 167.71(C = O), 175.52 (COOH) ppm. Anal. Calcd. for C_21_H_18_N_6_O_3_: C, 62.68; H, 4.51; N, 20.88 Found: C, 62.51; H, 4.71; N, 20.62.

#### 3-Methyl-2-{4-[(1-phenyl-1H-pyrazolo[3,4-d]pyrimidin-4-yl)amino]benzamido}butanoic acid (7c)

Yield: 80%. m.p. 324–326 °C; ^1^H NMR (500 MHz, DMSO-d_6_) *δ*: 0.96 (d, *J* = 24.0 Hz, 6H, 2 CH_3_), 1.17–1.25 (m, 1H, CH C_3_), 1.93 (s, 1H, NH), 4.03 (s, 1H, NH), 4.28 (d, *J* = 8.0 Hz, 1H, CH C_2_), 7.38 (t, *J* = 14.0 Hz, 1H, Ar-H), 7.59 (t, *J* = 12.0 Hz, 2H, Ar-H), 8.04 (d, *J* = 16.0 Hz, 4H, Ar-H), 8.22 (s, 2H, Ar-H), 8.66 (d, *J* = 16.0 Hz, 2H, Ar-H), 10.56 (s, 1H, COOH) ppm; ^13^C NMR (100 MHz, DMSO-d_6_) *δ*: 19.03 (2 CH_3_), 30.88 (CH, C_3_), 59.66 (CH, C_2_), 100.35 (Ar-C), 119.90 (2 Ar-C), 121.26(2 Ar-C), 126.55 (Ar-C), 127.47 (2 Ar-C), 128.34 (Ar-C), 129.37 (2 Ar-C), 136.25 (Ar-C), 139.40 (Ar-C), 144.90 (Ar-C), 152.55 (Ar-C), 152.87 (Ar-C), 154.72 (Ar-C), 167.85(C = O), 176.55 (COOH) ppm. Anal. Calcd. for C_23_H_22_N_6_O_3_: C, 64.17; H, 5.15; N, 19.52 Found: C, 64.33; H, 5.22; N, 19.60.

#### 3-Methyl-2-{4-[(1-phenyl-1H-pyrazolo[3,4-d]pyrimidin-4-yl)amino]benzamido}pentanoic acid (7d)

Yield: 74%. m.p. 338–3406 °C; ^1^H NMR (500 MHz, DMSO-d_6_) *δ*: 0.93 (d, *J* = 24.0 Hz, 3H, CH_3_ C_3_), 1.31 (t, *J* = 4.0 Hz, 3H, CH_3_ C_4_), 1.50–1.56 (m, 2H, CH_2_), 1.93 (s, 1H, NH), 1.95–1.99 (m, 1H, CH C_3_), 3.96 (s, 1H, NH), 4.38 (d, *J* = 5.0 Hz, 1H, CH C_2_), 7.39 (t, *J* = 8.0 Hz, 1H, Ar-H), 7.59 (t, *J* = 8.0 Hz, 2H, Ar-H), 8.05 (d, *J* = 8.0 Hz, 4H, Ar-H), 8.22 (s, 1H, Ar-H), 8.36 (s, 1H, Ar-H), 8.66 (d, *J* = 8.0 Hz, 2H, Ar-H), 10.53 (s, 1H, COOH) ppm; ^13^C NMR (100 MHz, DMSO-d_6_) *δ*:11.56 (CH_3_, C_4_), 16.27 (CH_3_, C_3_), 24.95 (CH_2_, C_4_), 35.26 (CH, C_3_), 58.62 (CH, C_2_), 100.31 (Ar-C), 121.24 (2 Ar-C), 121.55 (Ar-C), 122.25(Ar-C), 126.58 (Ar-C), 126.83 (Ar-C), 127.68 (Ar-C), 129.26 (2 Ar-C), 136.25 (Ar-C), 138.68 (Ar-C), 139.44 (Ar-C), 142.57 (Ar-C), 152.54 (Ar-C), 152.86 (Ar-C), 154.76 (Ar-C), 167.54(C = O), 176.17 (COOH) ppm. Anal. Calcd. for C_24_H_24_N_6_O_3_: C, 64.85; H, 5.44; N, 18.91 Found: C, 65.09; H, 5.58; N, 19.11.

#### 2-{4-[(1-Phenyl-1H-pyrazolo[3,4-d]pyrimidin-4-yl)amino]benzamido}pentanedioic acid (7e)

Yield: 89%. m.p. 375–377 °C; ^1^H NMR (500 MHz, DMSO-d_6_) *δ*: 1.22 (s, 1H, NH), 1.98–2.11 (q, *J* = 7.0 Hz, 2H, CH_2_ C_3_), 2.40 (t, *J* = 8.0 Hz, 2H, CH_2_ C_4_), 4.00 (s, 1H, NH), 4.43 (t, J = 12.0 Hz, 1H, CH C_2_), 7.40 (t, J = 4.0 Hz, 1H, Ar-H), 7.60 (t, *J* = 8.0 Hz, 2H, Ar-H), 7.97 (d, *J* = 8.0 Hz, 2H, Ar-H), 8.04 (d, *J* = 12.0 Hz, 2H, Ar-H), 8.22 (s, 1H, Ar-H), 8.24 (s, 1H, Ar-H), 8.59 (d, *J* = 8.0 Hz, 1H, Ar-H), 8.65 (m, 1H, Ar-H), 10.51 (s, 1H, COOH), 12.48 (s, 1H, COOH) ppm; ^13^C NMR (100 MHz, DMSO-d_6_) *δ*: 26.95 (CH_2_, C_3_), 30.57 (CH_2_, C_4_), 53.91 (CH, C_2_), 100.31(Ar-C), 119.95 (2 Ar-C), 121.23 (2 Ar-C), 126.52 (Ar-C), 127.44 (2 Ar-C), 128.35 (Ar-C), 129.30 (2 Ar-C), 136.25 (Ar-C), 139.40 (Ar-C), 144.86 (Ar-C), 152.55 (Ar-C), 152.87 (Ar-C), 154.66 (Ar-C), 167.80 (C = O), 175.47 (COOH, C_1_), 176.74 (COOH, C_5_) ppm. Anal. Calcd. for C_23_H_20_N_6_O_5_: C, 60.00; H, 4.38; N, 18.25 Found: C, 60.17; H, 4.52; N, 18.40.

#### 5-Guanidino-2-{4-[(1-phenyl-1H-pyrazolo[3,4-d]pyrimidin-4-yl)amino]benzamido}pentanoic acid (7f)

Yield: 78%. m.p. 404–406 °C; ^1^H NMR (500 MHz, DMSO-d_6_) *δ*: 0.36 (s, 2H, NH_2_ guanidine), 0.57 (s, 1H, NH guanidine), 0.79–0.85 (m, 2H, CH_2_ C_4_), 1.09–1.21 (q, *J* = 16.0 Hz, 2H, CH_2_ C_3_), 1.66 (t, *J* = 18.0 Hz, 2H, CH_2_ C_5_), 1.90 (s, 1H, NH), 3.69 (s, 1H, NH guanidine), 4.00 (s, 1H, NH), 4.38 (t, *J* = 4.0 Hz, 1H, CH C_2_), 7.37 (t, *J* = 8.0 Hz, 1H, Ar-H), 7.57 (t, *J* = 6.0 Hz, 2H, Ar-H), 8.00 (d, *J* = 8.0 Hz, 2H, Ar-H), 8.06 (d, *J* = 8.0 Hz, 2H, Ar-H), 8.19 (s, 1H, Ar-H), 8.21 (s, 1H, Ar-H), 8.64 (d, *J* = 12.0 Hz, 2H, Ar-H), 10.56 (s, 1H, COOH) ppm; ^13^C NMR (100 MHz, DMSO-d_6_) *δ*: 25.83 (CH_2_, C_4_), 29.53 (CH_2_, C_3_), 41.88 (CH_2_, C_5_), 55.18 (CH, C_2_), 100.31 (Ar-C), 119.90 (2 Ar-C), 121.25 (2 Ar-C), 126.53 (Ar-C), 127.47 (2 Ar-C), 128.35 (Ar-C), 129.28 (2 Ar-C), 136.29 (Ar-C), 139.40 (Ar-C), 144.88 (Ar-C), 152.55 (Ar-C), 152.88 (Ar-C), 154.73 (Ar-C), 157.16 (C = NH), 167.80 (C = O), 175.47 (COOH) ppm. Anal. Calcd. for C_24_H_25_N_9_O_3_: C, 59.13; H, 5.17; N, 25.86 Found: C, 59.30; H, 5.12; N, 25.99.

#### 3-Mercapto-2-{4-[(1-phenyl-1H-pyrazolo[3,4-d]pyrimidin-4-yl)amino]benzamido}propanoic acid (7g)

Yield: 90%. m.p. 348–350 °C; ^1^H NMR (500 MHz, DMSO-d_6_) *δ*: 1.18 (s, 1H, SH), 1.93 (s, 1H, NH), 3.13 (d, *J* = 4.0 Hz, 2H, CH_2_ C_3_), 4.01 (s, 1H, NH), 4.69 (t, *J* = 12.0 Hz, 1H, CH C_2_), 7.38 (t, *J* = 8.0 Hz, 1H, Ar-H), 7.57 (t, *J* = 8.0 Hz, 2H, Ar-H), 8.01 (d, *J* = 8.0 Hz, 4H, Ar-H), 8.20 (s, 2H, Ar-H), 8.62 (d, *J* = 8.0 Hz, 2H, Ar-H), 10.54 (s, 1H, COOH) ppm; ^13^C NMR (100 MHz, DMSO-d_6_) *δ*: 28.23 (CH_2_, C_3_), 57.11 (CH, C_2_), 100.33 (Ar-H), 119.96 (2 Ar-H), 121.26 (2 Ar-H), 126.59 (Ar-H), 127.43 (2 Ar-H), 128.38 (Ar-H), 129.37 (2 Ar-H), 136.25 (Ar-H), 139.40 (Ar-H), 144.87 (Ar-H), 152.54 (Ar-H), 152.87 (Ar-H), 154.70 (Ar-H), 167.80 (C = O), 176.32 (COOH) ppm. Anal. Calcd. for C_21_H_18_N_6_O_3_S: C, 58.05; H, 4.18; N, 19.34 Found: C, 58.14; H, 4.32; N, 19.49.

#### 4-(Methylthio)-2-{4-[(1-phenyl-1H-pyrazolo[3,4-d]pyrimidin-4-yl)amino]benzamido}butanoic acid (7h)

Yield: 83%. m.p. 344–346 °C; ^1^HNMR (500 MHz, DMSO-d_6_) *δ*: 1.15–1.23 (q, *J* = 12.0 Hz, 2H, CH_2_ C_3_), 1.93 (s, 1H, NH), 2.08 (s, 3H, CH_3_), 2.60 (t, *J* = 6.0 Hz, 2H, CH_2_ C_4_), 4.00 (s, 1H, NH), 4.50 (t, *J* = 14.0 Hz, 1H, CH C_2_), 7.43 (t, *J* = 24.0 Hz, 1H, Ar-H), 7.58 (t, *J* = 20.0 Hz, 2H, Ar-H), 8.04 (d, *J* = 20.0 Hz, 4H, Ar-H), 8.22 (s, 2H, Ar-H), 8.65 (d, *J* = 12.0 Hz, 2H, Ar-H), 10.55 (s, 1H, COOH) ppm; ^13^C NMR (100 MHz, DMSO-d_6_) *δ*: 14.64 (CH_3_), 29.91 (CH_2_, C_4_), 31.61 (CH_2_, C_3_), 53.61 (CH, C_2_), 100.34 (Ar-H), 119.97 (2 Ar-H), 121.28 (2 Ar-H), 126.53 (Ar-H), 127.47 (2 Ar-H), 128.35 (Ar-H), 129.38 (2 Ar-H), 136.34 (Ar-H), 139.45 (Ar-H), 144.93 (Ar-H), 152.58 (Ar-H), 152.91 (Ar-H), 154.72 (Ar-H), 167.80 (C = O), 175.47 (COOH) ppm. Anal. Calcd. for C_23_H_22_N_6_O_3_S: C, 59.73; H, 4.79; N, 18.17 Found: C, 59.89; H, 4.90; N, 18.39.

#### 3-(1h-Imidazol-4-yl)-2-{4-[(1-phenyl-1H-pyrazolo[3,4-d]pyrimidin-4-yl)amino]benzamido}-propanoic acid (7i)

Yield: 82%. m.p. 381–383 °C; ^1^H NMR (500 MHz, DMSO-d_6_) *δ*: 1.16 (s, 1H, NH imidazole), 1.93 (s, 1H, NH), 3.13 (d, *J* = 12.0 Hz, 2H, CH_2_), 4.00 (s, 1H, NH), 4.62 (t, *J* = 12.0 Hz, 1H, CH C_2_), 6.91 (s, 1H, CH, imidazole C_5_), 7.37 (t, *J* = 20.0 Hz, 1H, Ar-H), 7.57 (t, *J* = 12.0 Hz, 2H, Ar-H), 7.92 (s, 1H, CH, imidazole C_2_), 8.03 (d, *J* = 16.0 Hz, 4H, Ar-H), 8.21 (s, 2H, Ar-H), 8.64 (d, *J* = 16.0 Hz, 2H, CH_2_), 10.56 (s, 1H, COOH) ppm; ^13^C NMR (100 MHz, DMSO-d_6_) *δ*: 29.09 (CH_2_, C_3_), 50.83 (CH, C_2_),100.31 (Ar-C),118.23 (imidazole C_5_),119.99 (2 Ar-H),121.26 (2 Ar-H),126.59 (Ar-H),127.49 (2 Ar-H),128.37 (Ar-H),129.34 (2 Ar-H),131.23 (imidazole C_4_),134.60 (imidazole C_2_), 136.33 (Ar-H), 139.47 (Ar-H), 144.91 (Ar-H), 152.53 (Ar-H), 152.89 (Ar-H), 154.72 (Ar-H), 167.80 (C = O), 174.39 (COOH) ppm. Anal. Calcd. for C_24_H_20_N_8_O_3_: C, 61.53; H, 4.30; N, 23.92 Found: C, 61.42; H, 4.21; N, 23.88.

#### 1-{4-[(1-Phenyl-1H-pyrazolo[3,4-d]pyrimidin-4-yl)amino]benzoyl}pyrrolidine-2-carboxylic acid (7j)

Yield: 77%. m.p. 342–344 °C; ^1^HNMR (500 MHz, DMSO-d_6_) *δ*: 1.18–1.28 (m, 2H, CH_2_, C_4_ pyrrolidine), 2.25–2.30 (q, 2H, *J* = 6.7 Hz, CH_2_, C_3_ pyrrolidine), 3.40 (t, *J* = 8.0 Hz, 2H, CH_2_, C_5_ pyrrolidine), 4.01 (s, 1H, NH), 4.42 (t, *J* = 8.0 Hz, 1H, CH, C_2_ pyrrolidine), 7.39 (t, *J* = 16.0 Hz, 1H, Ar-H), 7.59 (t, *J* = 18.0 Hz, 2H, Ar-H), 8.04 (d, *J* = 20.0 Hz, 4H, Ar-H), 8.22 (s, 2H, Ar-H), 8.66 (d, *J* = 20.0 Hz, 2H, Ar-H), 10.57 (s, 1H, COOH) ppm; ^13^C NMR (100 MHz, DMSO-d_6_) *δ*: 24.63 (C_4_, pyrrolidine), 29.06 (C_3_, pyrrolidine),45.62 (C_5_, pyrrolidine), 61.85 (C_2_, pyrrolidine), 100.31 (Ar-H), 119.65 (2 Ar-H), 121.28 (2 Ar-H), 126.58 (Ar-H), 127.40 (2 Ar-H), 129.36 (2 Ar-H), 129.72 (Ar-H), 136.38 (Ar-H), 139.40 (Ar-H), 144.40 (Ar-H), 152.58 (Ar-H), 152.86 (Ar-H), 154.76 (Ar-H), 170.60 (C = O), 175.14 (COOH) ppm. Anal. Calcd. for C_23_H_20_N_6_O_3_: C, 64.48; H, 4.71; N, 19.62 Found: C, 64.56; H, 4.86; N, 19.79.

#### 3-(1h-Indol-3-yl)-2-{4-[(1-phenyl-1H-pyrazolo[3,4-d]pyrimidin-4-yl)amino]benzamido}propanoic acid (7k)

Yield: 87%. m.p. 398–400 °C; ^1^HNMR (500 MHz, DMSO-d_6_) *δ*: 1.18 (s, 1H, NH), 3.33 (d, *J* = 40.0 Hz, 2H, CH_2_ C_3_), 4.01 (s, 1H, NH), 4.69 (t, *J* = 4.0 Hz, 1H, CH C_2_), 7.03 (d, *J* = 12.0 Hz, 1H, Ar-H), 7.38 (t, *J* = 12.0 Hz, 2H, Ar-H), 7.59 (t, *J* = 14.0 Hz, 3H, Ar-H), 7.92 (s, 1H, Ar-H), 8.04 (d, *J* = 12.0 Hz, 4H, Ar-H), 8.22 (s, 2H, Ar-H), 8.65 (d, *J* = 16.0 Hz, 3H, Ar-H), 10.56 (s, 1H, NH indole), 10.86 (s, 1H, COOH) ppm; ^13^C NMR (100 MHz, DMSO-d_6_) *δ*: 28.42 (CH_2_, C_3_), 51.98 (CH, C_2_), 100.32 (Ar-H), 108.54 (indole C_3_), 111.57 (Ar-H), 119.49 (Ar-H), 119.99 (2 Ar-H), 120.12 (Ar-C), 121.24 (2 Ar-H), 121.69 (Ar-H), 123.42 (indole C_2_), 126.52 (Ar-H), 127.47 (2 Ar-H), 127.95 (Ar-H), 128.37 (Ar-H), 129.37 (2 Ar-H), 136.28 (Ar-H), 137.29 (Ar-H), 139.40 (Ar-H), 144.94 (Ar-H), 152.56 (Ar-H), 152.90 (Ar-H), 154.73 (Ar-H), 167.79 (C = O), 174.39 (COOH) ppm. Anal. Calcd. for C_29_H_23_N_7_O_3_: C, 67.30; H, 4.48; N, 18.94 Found: C, 67.44; H, 4.60; N, 19.09.

#### 3-Phenyl-2-{4-[(1-phenyl-1H-pyrazolo[3,4-d]pyrimidin-4-yl)amino]benzamido}propanoic acid (7l)

Yield: 84%. m.p. 387–389 °C; ^1^H NMR (500 MHz, DMSO-d_6_) *δ*: 1.92 (s, 1H, NH), 3.25 (d, *J* = 4.0 Hz, 2H, CH_2_ C_3_), 4.00 (s, 1H, NH), 4.60 (t, *J* = 8.0 Hz, 1H, CH C_2_), 7.17 (t, *J* = 8.0 Hz, 1H, Ar-H), 7.26 (t, *J* = 8.0 Hz, 1H, Ar-H), 7.33 (d, *J* = 8.0 Hz, 1H, Ar-H), 7.38 (t, *J* = 8.0 Hz, 1H, Ar-H), 7.58 (t, *J* = 8.0 Hz, 3H, Ar-H), 7.87 (d, *J* = 8.0 Hz, 1H, Ar-H), 8.01 (d, *J* = 8.0 Hz, 2H, Ar-H), 8.07 (d, *J* = 8.0 Hz, 2H, Ar-H), 8.21 (s, 1H, Ar-H), 8.23 (s, 1H, Ar-H), 8.62 (d, *J* = 12.0 Hz, 1H, Ar-H), 8.67 (d, *J* = 8.0 Hz, 1H, Ar-H), 10.58 (s, 1H, COOH) ppm; ^13^C NMR (100 MHz, DMSO-d_6_) *δ*: 37.05 (CH_2_, C_3_), 55.97 (CH, C_2_), 100.35 (Ar-H), 119.94 (2 Ar-H), 121.28 (2 Ar-H), 126.55 (Ar-H), 127.15 (Ar-H), 127.51 (2 Ar-H), 128.37 (Ar-H), 129.05 (2 Ar-H), 129.34 (2 Ar-H), 129.38 (2 Ar-H), 136.28 (Ar-H), 137.68 (Ar-H), 139.42 (Ar-H), 144.90 (Ar-H), 152.54 (Ar-H), 152.92 (Ar-H), 154.70 (Ar-H), 167.82 (C = O), 174.49 (COOH) ppm. Anal. Calcd. for C_27_H_22_N_6_O_3_: C, 67.77; H, 4.63; N, 17.56 Found: C, 67.90; H, 4.86; N, 17.70.

#### 3-(4-Hydroxyphenyl)-2-{4-[(1-phenyl-1H-pyrazolo[3,4-d]pyrimidin-4-yl)amino]benzamido}-propanoic acid (7m)

Yield: 80%. m.p. 401–403 °C; ^1^H NMR (500 MHz, DMSO-d_6_) *δ*: 1.24 (s, 1H, OH), 1.92 (s, 1H, NH), 3.00 (d, *J* = 4.0 Hz, 2H, CH_2_ C_3_), 4.01 (s, 1H, NH), 4.43 (t, *J* = 16.0 Hz, 1H, CH C_2_), 6.62 (d, *J* = 8.0 Hz, 1H, Ar-H), 7.07 (t, *J* = 6.0 Hz, 1H, Ar-H), 7.19 (t, *J* = 6.0 Hz, 2H, Ar-H), 7.48 (d, *J* = 8.0 Hz, 3H, Ar-H), 7.94 (d, *J* = 8.0 Hz, 4H, Ar-H), 8.21 (s, 2H, Ar-H), 8.65 (d, *J* = 11.0 Hz, 2H, Ar-H), 10.59 (s, 1H, COOH) ppm; ^13^C NMR (100 MHz, DMSO-d_6_) *δ*: 37.07 (CH_2_, C_3_), 55.99 (CH, C_2_), 100.35 (Ar-H), 115.90 (2 Ar-H), 119.97 (2 Ar-H), 121.24 (2 Ar-H), 126.52 (Ar-H), 127.49 (2 Ar-H), 128.37 (Ar-H), 129.29 (2 Ar-H), 129.74 (Ar-H), 130.62 (2 Ar-H), 136.25 (Ar-H), 139.39 (Ar-H), 144.93 (Ar-H), 152.54 (Ar-H), 152.88 (Ar-H), 154.67 (Ar-H), 155.93 (Ar-H), 167.80 (C = O), 174.49 (COOH) ppm. Anal. Calcd. for C_27_H_22_N_6_O_4_: C, 65.58; H, 4.48; N, 16.99 Found: C, 65.69; H, 4.63; N, 17.14.

### Biological evaluation

#### Cytotoxicity screening using SRB assay

Sulforhodamine B (SRB) assay was employed to assay *in-vitro* antitumor activities of all the synthesised compounds as describer previously^11–14^. Six human cancer cell lines, human prostate cancer (PC-3), pancreatic human cancer cell lines (BxPC-3), colorectal carcinoma (HCT-116), human hepatocellular carcinoma (HepG-2), cervical carcinoma (HeLa), and mammary gland breast cancer (MCF-7), besides normal immortalised pancreatic cell line (HPDE) were used in the evaluation of antitumor activity. The cell lines were obtained from the American Type Culture Collection (Rockville, USA).

#### Elisa assay of human DHFR enzyme

Enzyme-linked immunosorbent assay (ELISA) assay technique was used to evaluate the anti-DHFR effects of all the synthesised compounds on as described previously^15^. The DHFR ELISA kit (MyBioSource, MBS9312476 San Diego, CA, USA) was used according to the manufacturer instructions. Methotrexate (MTX) was used as positive control, and a standard curve was plotted to detect IC_50_. The test was repeated in triplicate, and the significance was assessed by one-way ANOVA test followed by Tukey’s posttest, where *p* < 0.05 was considered significance.

#### Molecular modeling

**Figure 10.**
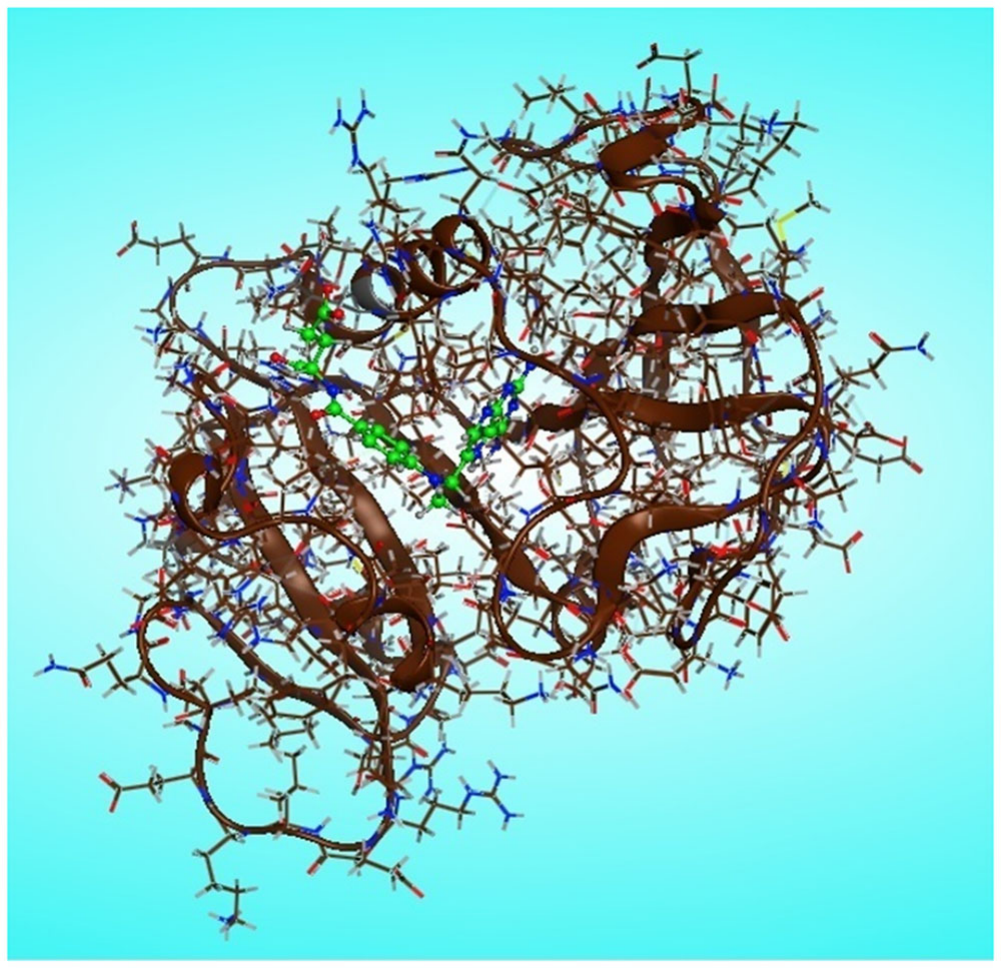
Chain A of DHFR enzyme with (green) methotrexate/NADPH complex ligand in the active site.

Molecular modelling calculations were carried out using molecular operating environment MOE version 2014.0901 (Chemical Computing Group Inc. software). The most stable conformers of newly synthesised pyrazolo[3,4-*d*]pyrimidine derivatives **6** and **7a–m** were docked into the binding pocket of DHFR-binding domain which is in tertiary complex with dihydro-nicotinamide-adenine-dinucleotide phosphate (NADPH) and methotrexate (MTX), (pdb code ID: 1U72)^18^. The three-dimensional structures of the novel prepared pyrazolo[3,4-*d*]pyrimidine derivatives, in their neutral forms, were built using the builder interface of the MOE software. Besides the referred enzymes were obtained from the research collaborator for structural bioinformatics Protein Data Bank (RCSB-PDB), where the hydrogens were added after releasing the backbone and side chain of amino acid residues into the secondary structures, and then the enzymes structure was subjected to a refinement protocol by automatic connect and type to correct the loosed bonds during X-ray crystallography followed by protein potential fixation where the constraints on the enzyme were gradually removed and minimised until the RMSD gradient was 0.01 kcal/mol A° as shown in Figure 10. The active site of the utilised enzymes in this study was detected using a radius of 10.0 A° around MTX. Conformational analysis of the newly synthesised pyrazolo[3,4-*d*]pyrimidine compounds was performed using MMFF94 force field with root mean square (RMS) gradient of 0.01 kcal/mol A°. The flexible alignment of our novel compounds was done using the flexible alignment tool of the MOE program adjusting the energy cut off to 15 kcal/mol, and RMSD tolerance to 0.5. A complete study was done for each ligand to check its fitting in the active site of the enzyme and to identify the different binding interactions involved in its fitting using MOE program software.

#### Cell apoptosis assay

Flow cytometric analysis using Annexin-V-fluoresce in isothiocyanate (FITC) and propidium iodide (PI) staining kit (BD Pharmingen, San Diego, USA) was performed to assess the apoptosis. At 70% confluence, the cells were treated with compound **7f** at different concentrations of (0–8 μmol/l) for 24 h. Then after trypsinization of cells, washing with PBS and centrifugation, the cells were mixed with FITC and PI in binding buffer. Becton Dickinson (Franklin Lakes, NJ, USA) was used to do the flow cytometry analysis^16^^,^^17^.

#### Assessment of the compound 7f effect on the caspase-3/7, Bax and Bcl2 activities

The apoptotic effect of compound **7f** on breast cancer cell line MCF-7 were evaluated by quantification of Bax, caspase 3/7, and Bcl-2 using Bax ELISA Kit ab199080 (ABCAM, Cambridge, UK), Caspase-Glo 3/7 assay kits (Promega, Fitchburg, MA, USA), and Bcl-2 ELISA Kit ab119506 (ABCAM, Cambridge, UK), according the instructions of manufacturers^11^^,^^14^. The cells were treated with compounds **7f** (½ IC_50_, IC_50_, or 2 ×IC_50_), and the activities were shown as a percentage change from the untreated control. The experiments were performed in triplicate applying two-way ANOVA test followed by Bonferroni posttest to evaluate the significance (*p* < 0.05).

#### Western blotting

The effect of compound **7f** on the expression of DHFR, Casp3, Bax, and Bcl-2 proteins in MCF-7 cancer cells was evaluated by western blotting as previously performed^20–22^. The cancer cells treated or untreated were collected were harvested and blotted on SDS-polyacrylamide gel and separated by Cleaver electrophoresis unit (Cleaver, UK), transferred onto polyvinylidene fluoride (PVDF) membranes (Millipore, USA) for 30 min using a Semi-dry Electroblotter (Biorad, USA) at 2.5 A and 25 V for 30 min. The membranes were blocked with 5% non-fat dry milk in TBS-T for 2 h at RT and then incubated overnight at 4 °C with anti-DHFR, anti-Bax, anti-Bcl-2, and anti-β-actin primary antibodies (abcam, Cambridge, UK). Finally, the horse radish peroxidase (HRP)-linked secondary antibody (Agilent, Santa Clara, CA, USA) was added for 1 h at room temperature. After washing, the chemiluminescent Western ECL substrate (Perkin Elmer, Waltham, MA, USA) was applied to the blot and then incubated for 1 min with a mixture of equal volumes from ECL solution A and ECL solution B. The chemiluminescent signals were captured using a CCD camera-based imager (Chemi Doc imager, Biorad, USA), and the bands intensities were then measured by Image *J* software program. Protein-sized markers were used in all gels to localise the gel transfer regions for specific proteins and determine the transfer efficiency. The experiments were conducted in triplicate. For colorimetric assays two-way ANOVA test followed by Bonferroni posttest were used, while for quantification expression on western blot, student-*t*-test was used (significance *p* < 0.05).

## Supplementary Material

Supplemental Material
