# Supplementary material for: Design, synthesis and antitumor evaluation of novel pyrazolo[3,4-d]pyrimidines incorporating different amino acid conjugates as potential DHFR inhibitors
Source: J Enzyme Inhib Med Chem. 2022 Nov 16;38(1):203–15. doi: 10.1080/14756366.2022.2142786 (PMC9673804; doi:10.1080/14756366.2022.2142786)
Supplement: Supplemental Material [file IENZ_A_2142786_SM2178.pdf]

Supporting Information

Design, Synthesis and Antitumor Evaluation of Novel Pyrazolo[3,4-*d*]pyrimidines Incorporating Different Amino acid Conjugates as Potential DHFR Inhibitors

Ibrahim M. Salem[a,*], Samia M. Mostafa[a], Ismail Salama[a], Osama I. El-Sabbagh[b], Wael A. H. Hegazy[c,d], Tarek S. Ibrahim[e,f,*]

[a] *Medicinal Chemistry Department, Faculty of Pharmacy, Suez Canal University, Ismailia 41522, Egypt*
[b] *Medicinal Chemistry Department, Faculty of Pharmacy, Zagazig University, Zagazig 44519, Egypt.*
[c] *Department of Microbiology and Immunology, Faculty of Pharmacy, Zagazig University, Zagazig 44519, Egypt.*
[d] *Pharmacy Program, Department of Pharmaceutical Sciences, Oman College of Health Sciences, Muscat 113, Oman.*
[e] *Department of Pharmaceutical Chemistry, Faculty of Pharmacy, King Abdulaziz University, Jeddah, 21589, Saudi Arabia.*
[f] *Department of Pharmaceutical Organic Chemistry, Faculty of Pharmacy, Zagazig University, Zagazig, 44519, Egypt.*

CONTACT: Ibrahim M. Salem dr_ibrahim_m@yahoo.com *Medicinal Chemistry Department, Faculty of Pharmacy, Suez Canal University, Ismailia 41522, Egypt* **;** Tarek S. Ibrahim; tmabrahem@kau.edu.sa *Department of Pharmaceutical Chemistry, Faculty of Pharmacy, King Abdulaziz University, Jeddah, 21589, Saudi Arabia.*

- ^{1}HNMR and ^{13}C NMR for compounds **6** and **7a-m.**
- Raw data, tables and images for Biological activities

^{1}H NMR and ^{13}C NMR of compound (6)

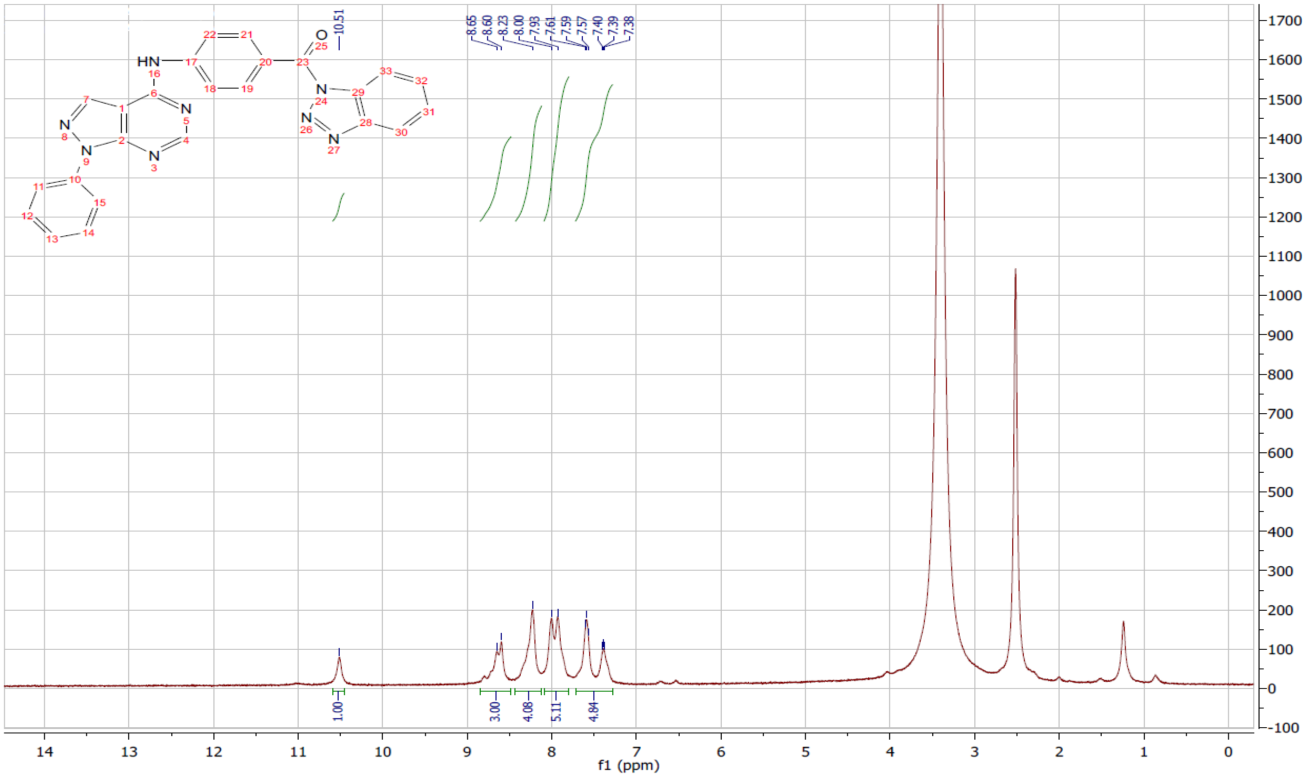

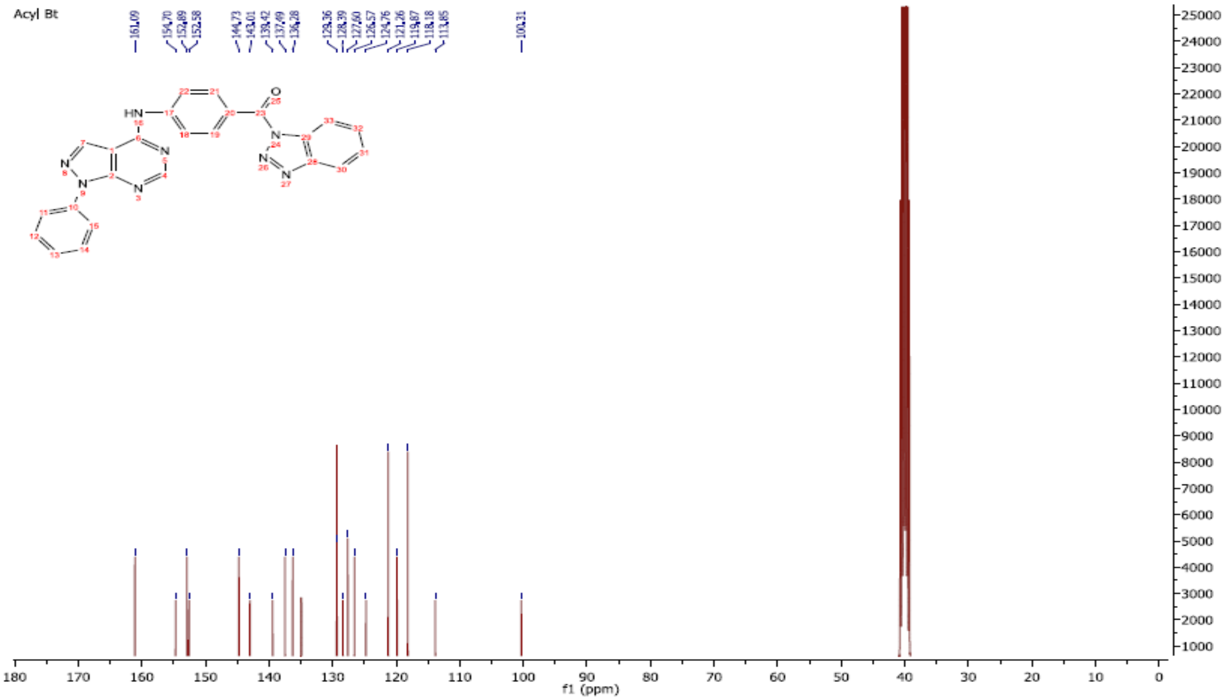

^{1}H NMR and ^{13}C NMR of compound (7_a)

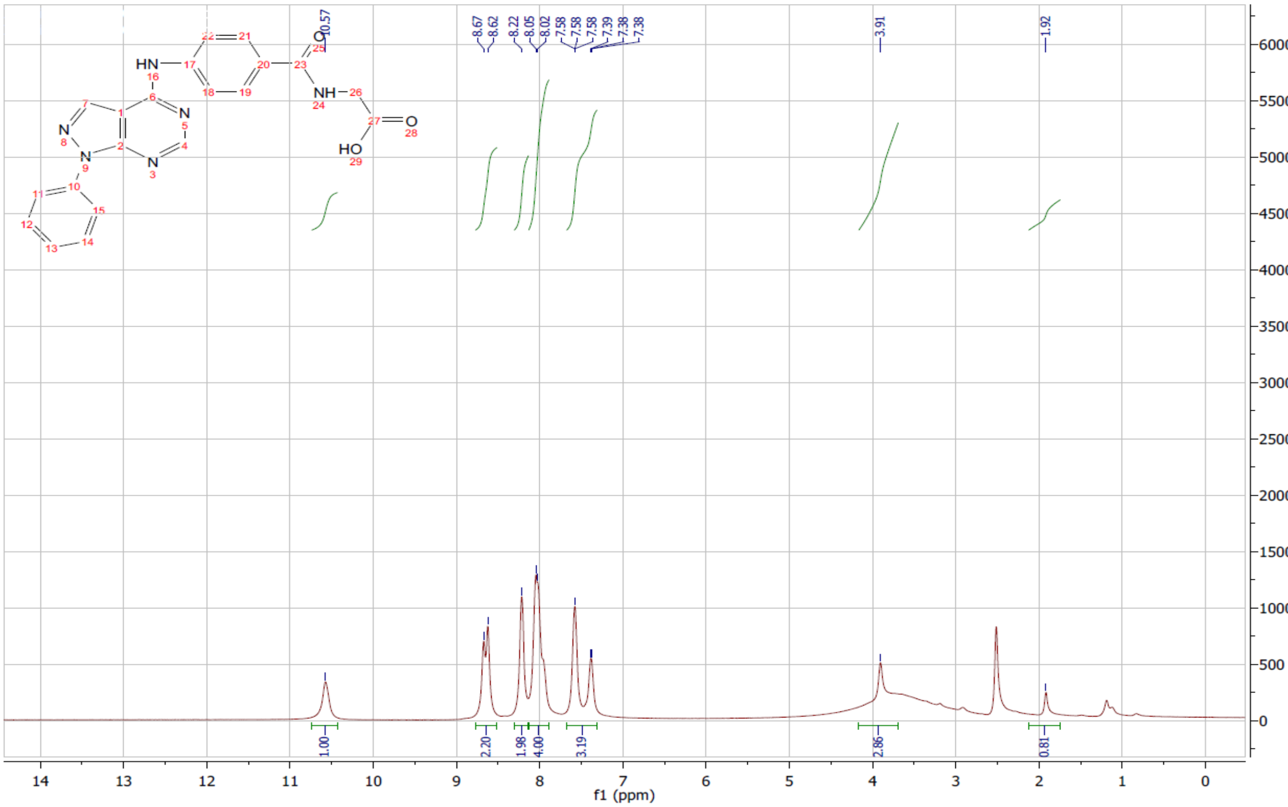

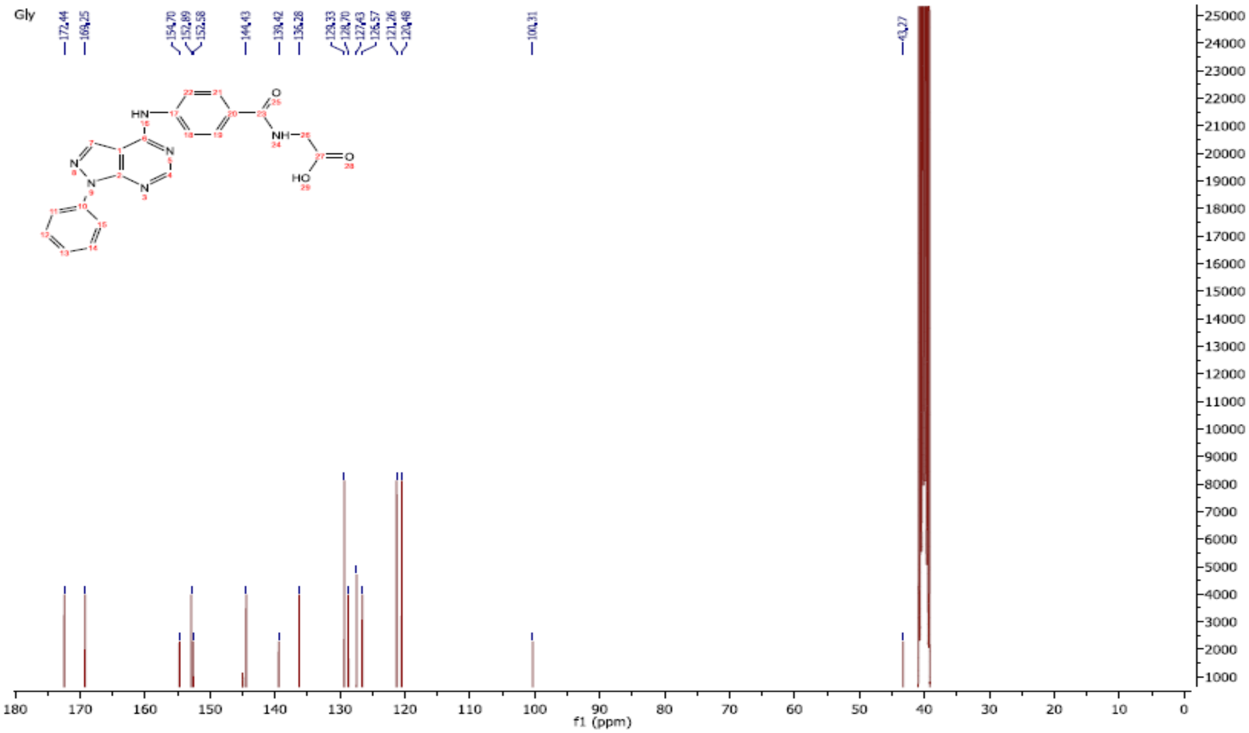

^{1}H NMR and ^{13}C NMR of compound (7$_b$)

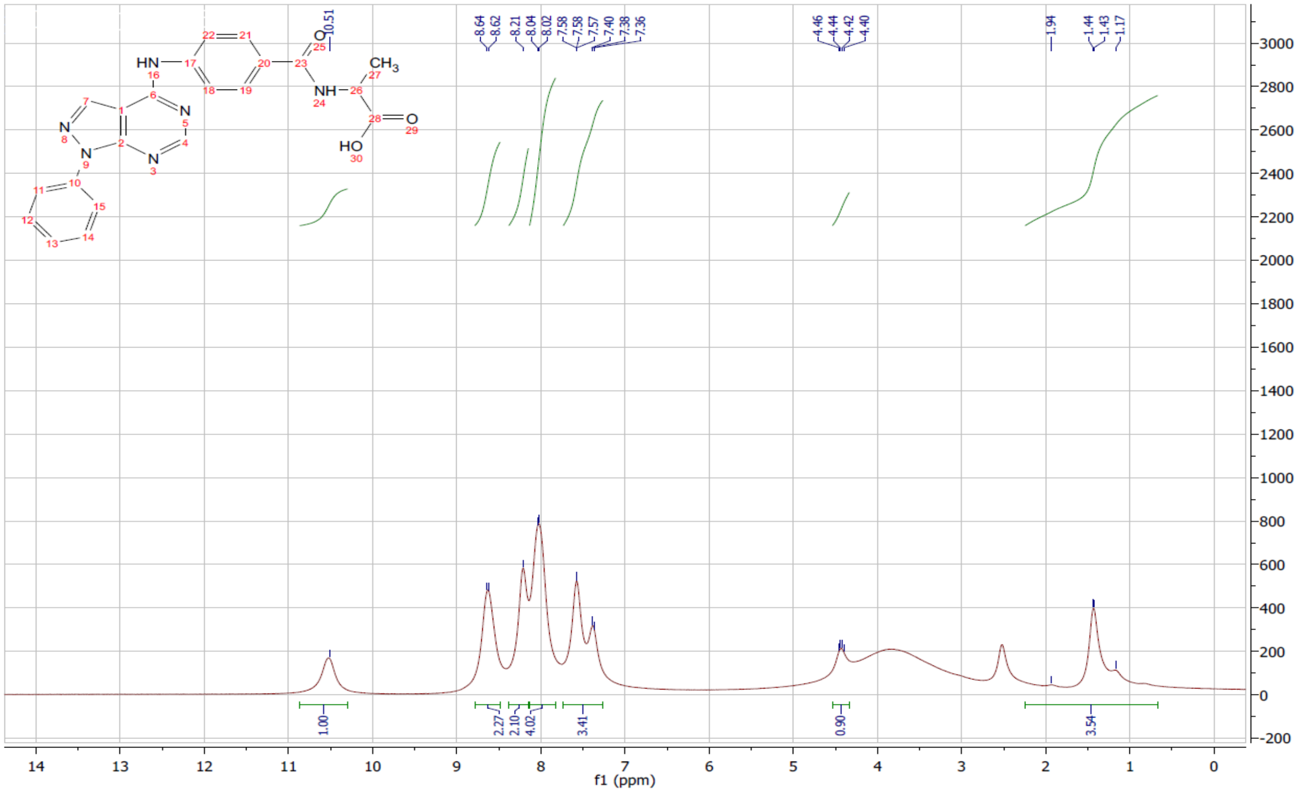

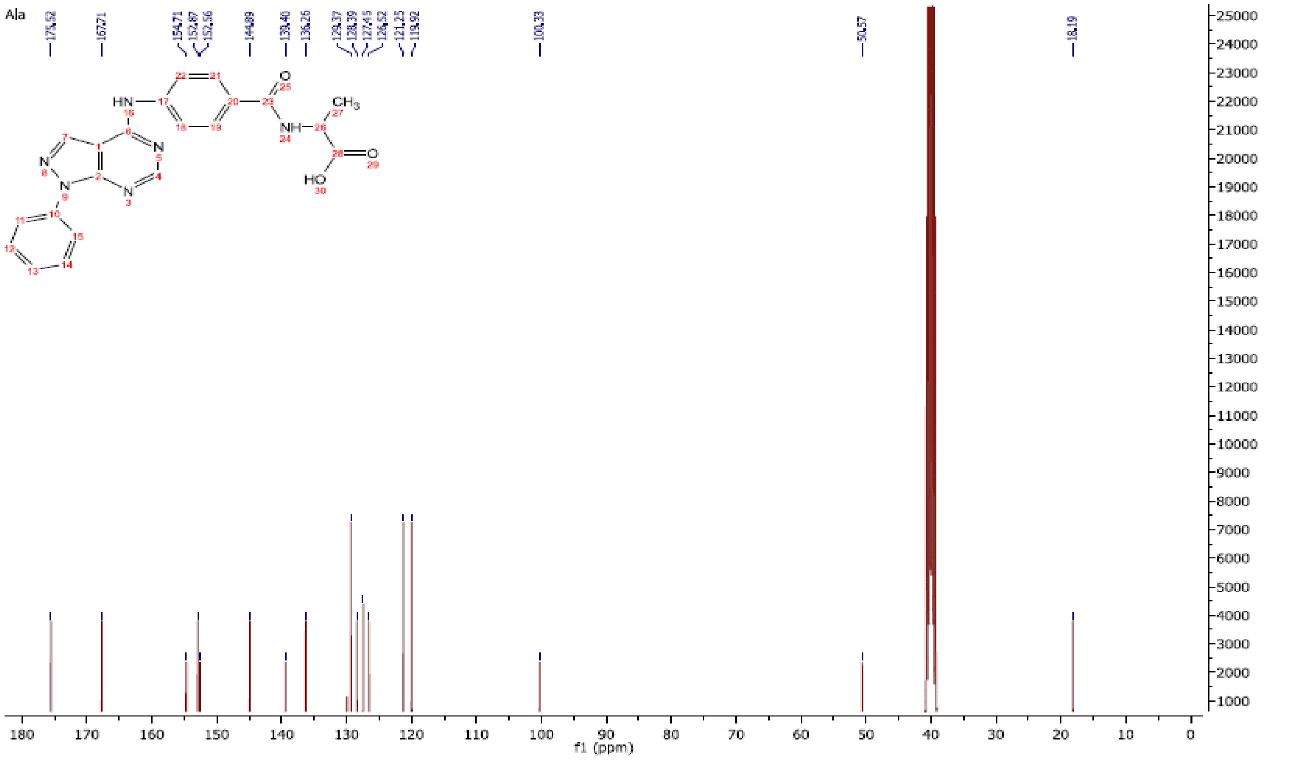

^{1}H NMR and ^{13}C NMR of compound (7_c)

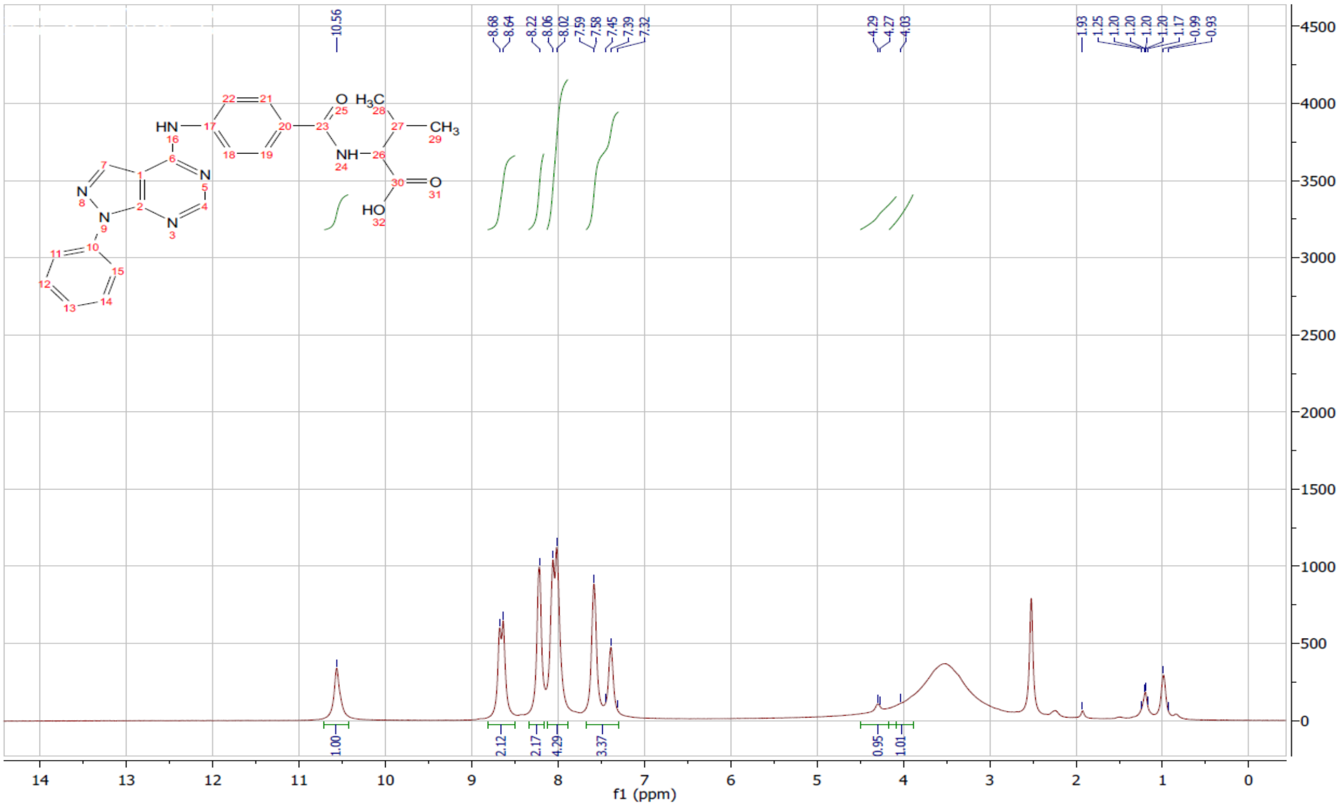

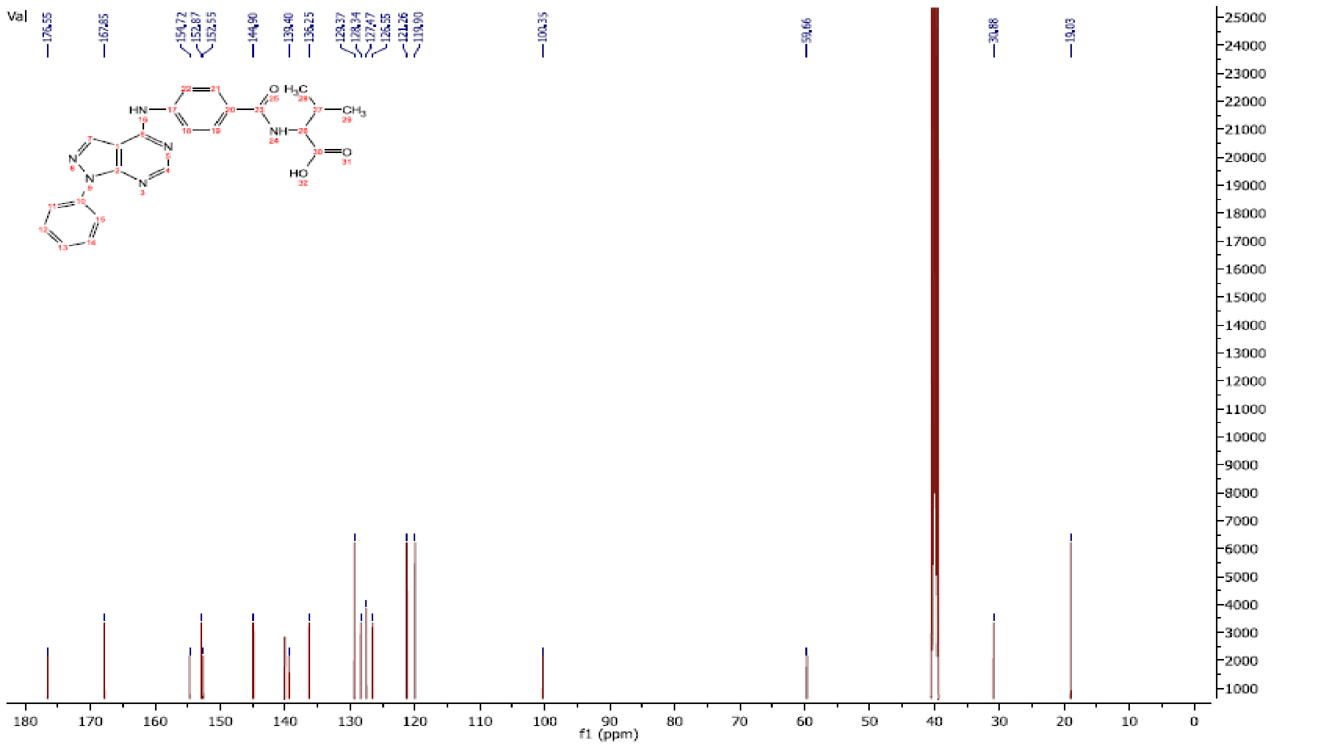

^{1}H NMR and ^{13}C NMR of compound (7_d)

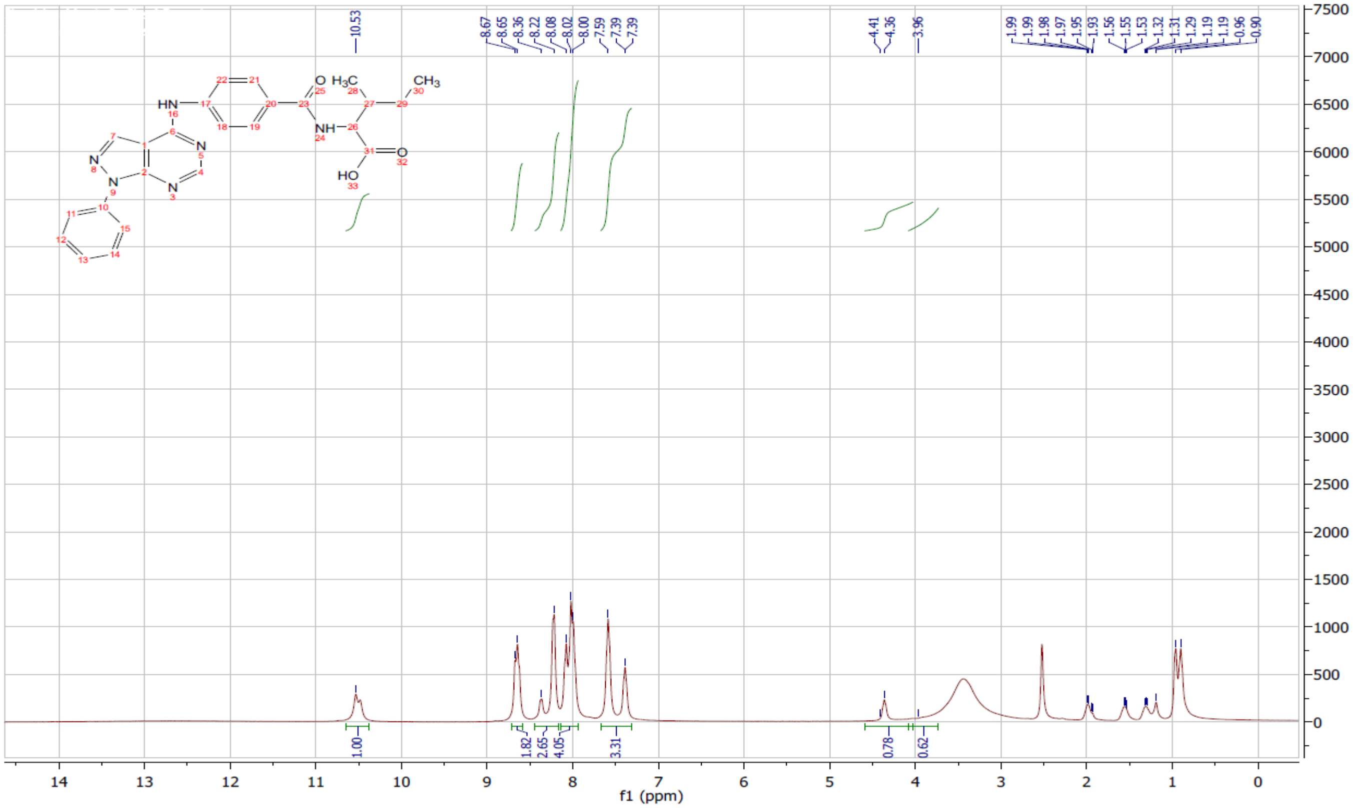

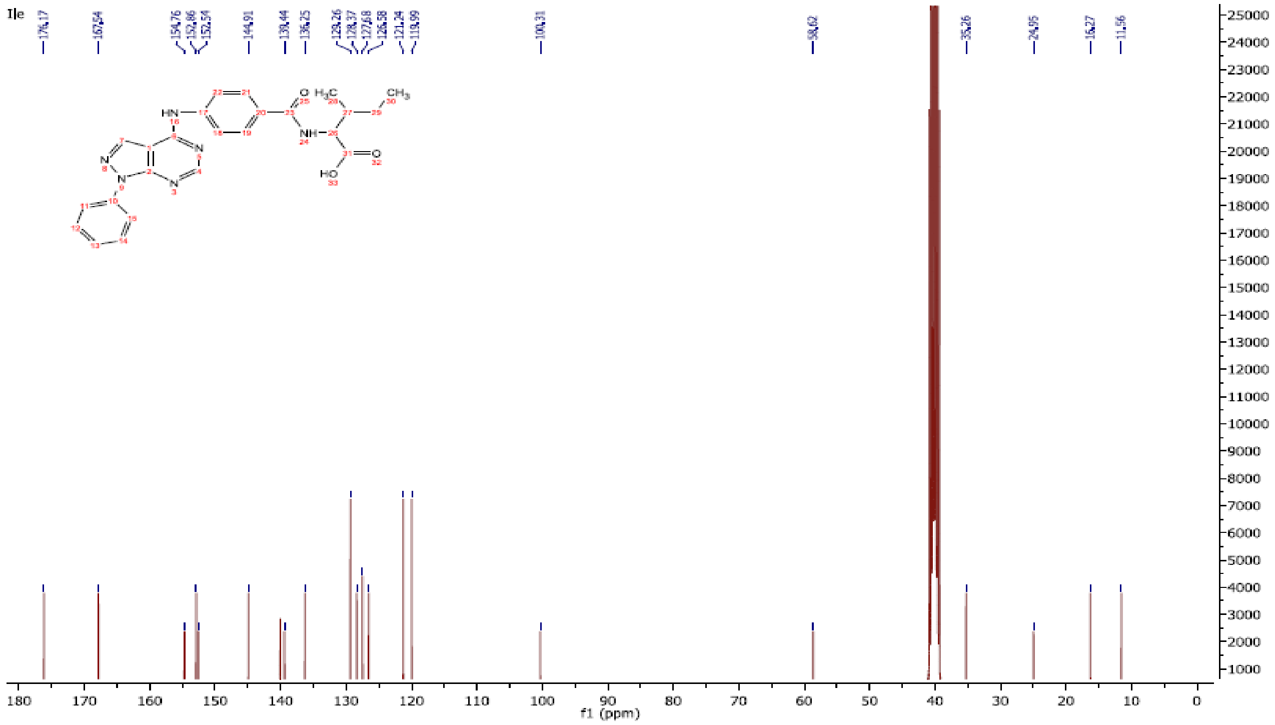

^{1}H NMR and ^{13}C NMR of compound (7_e)

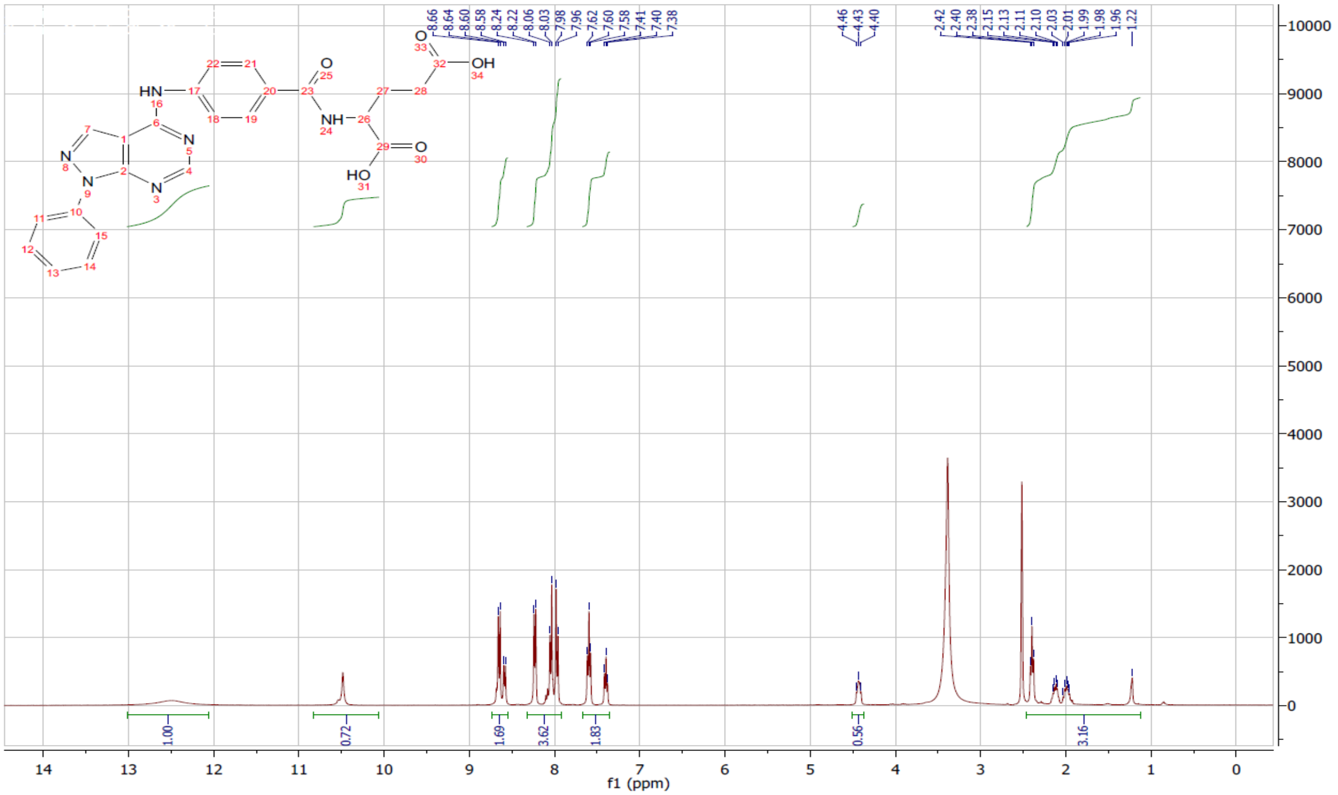

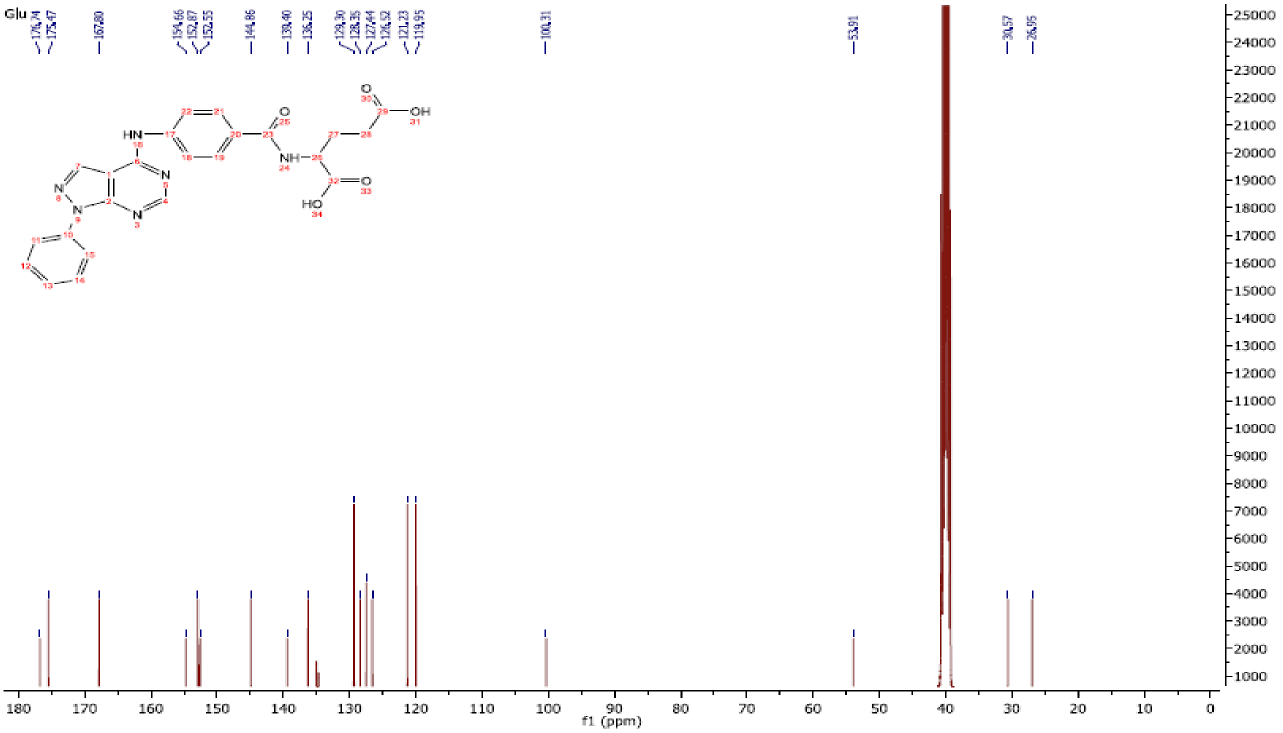

^{1}H NMR and ^{13}C NMR of compound (7f)

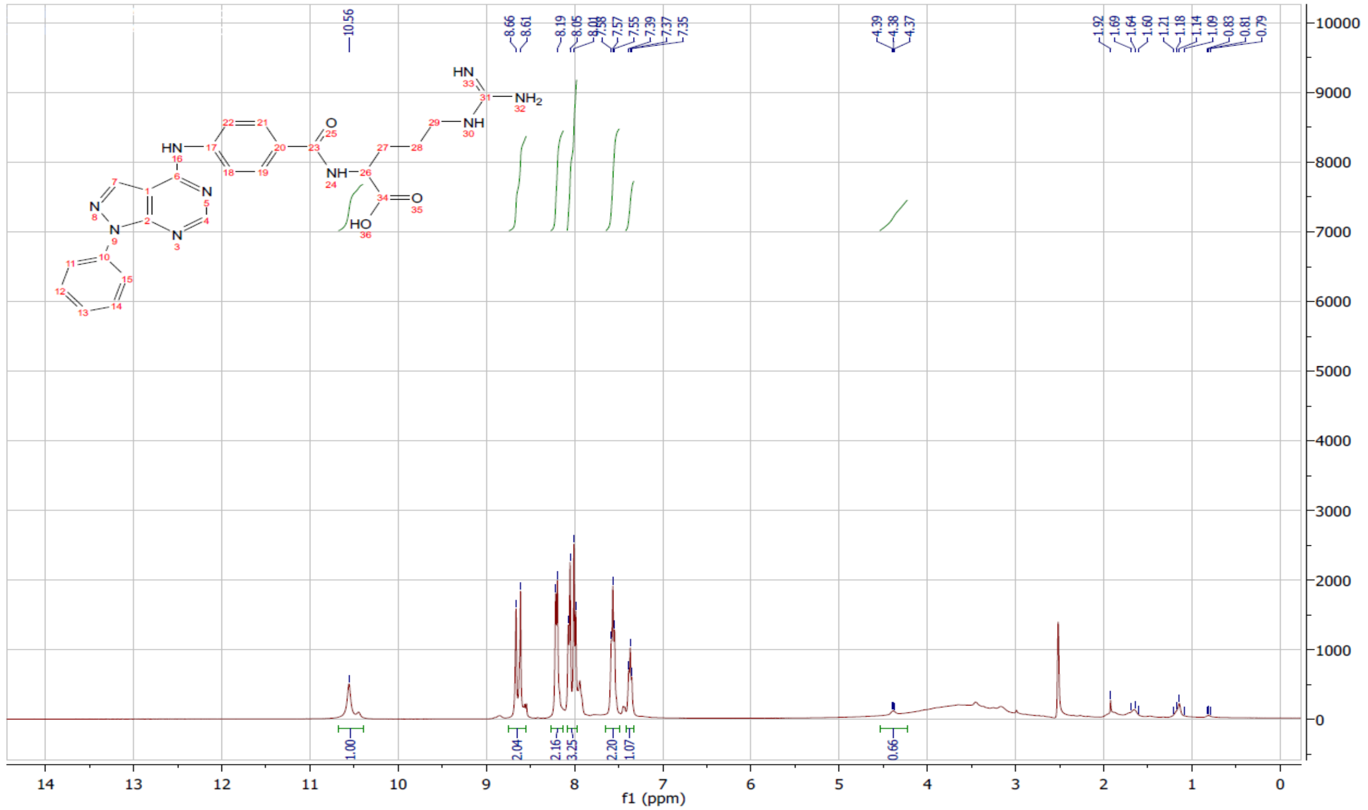

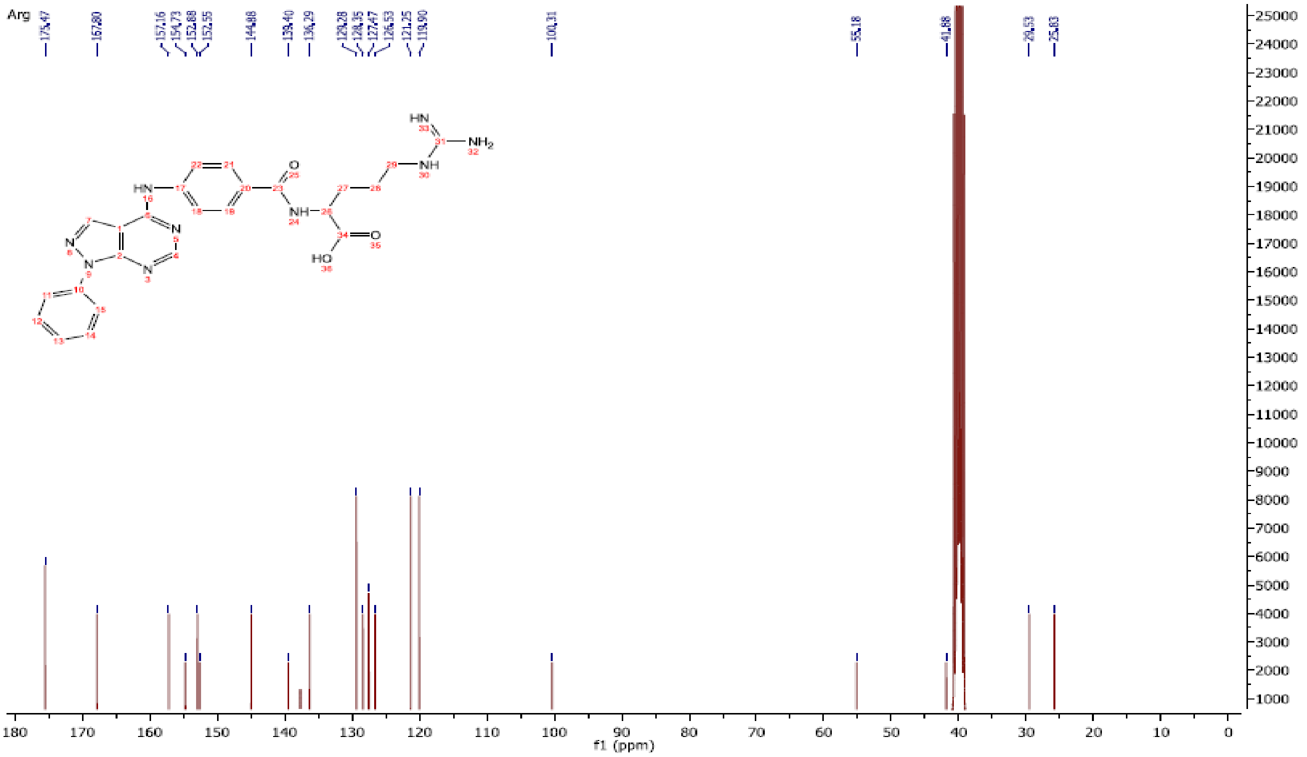

^{1}H NMR and ^{13}C NMR of compound (7_g)

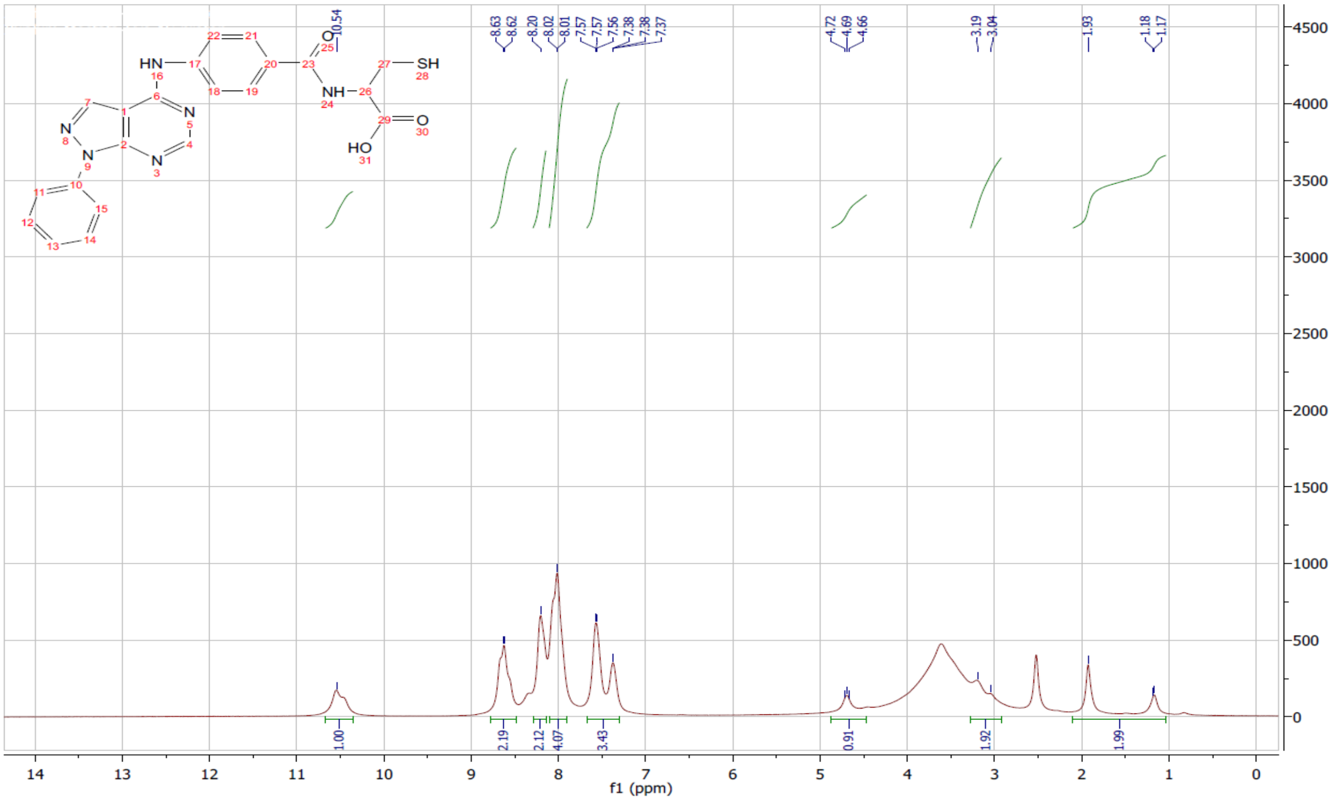

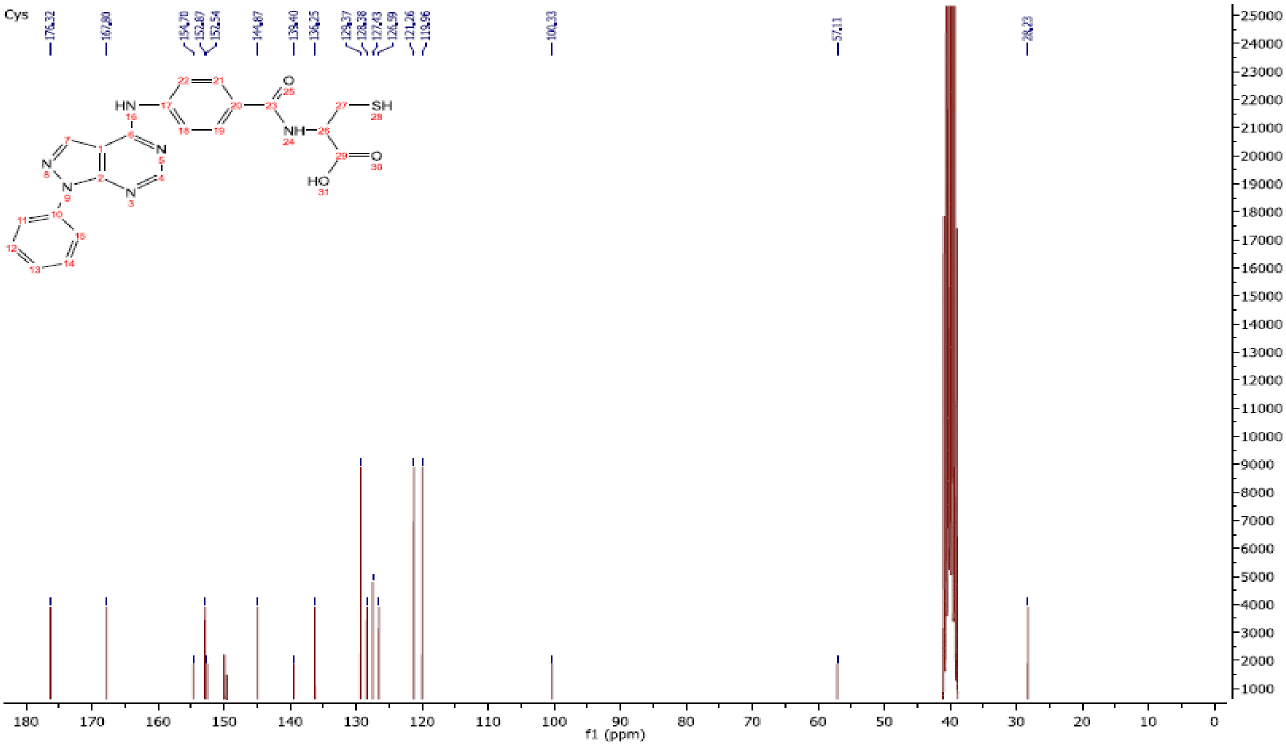

^{1}H NMR and ^{13}C NMR of compound (7h)

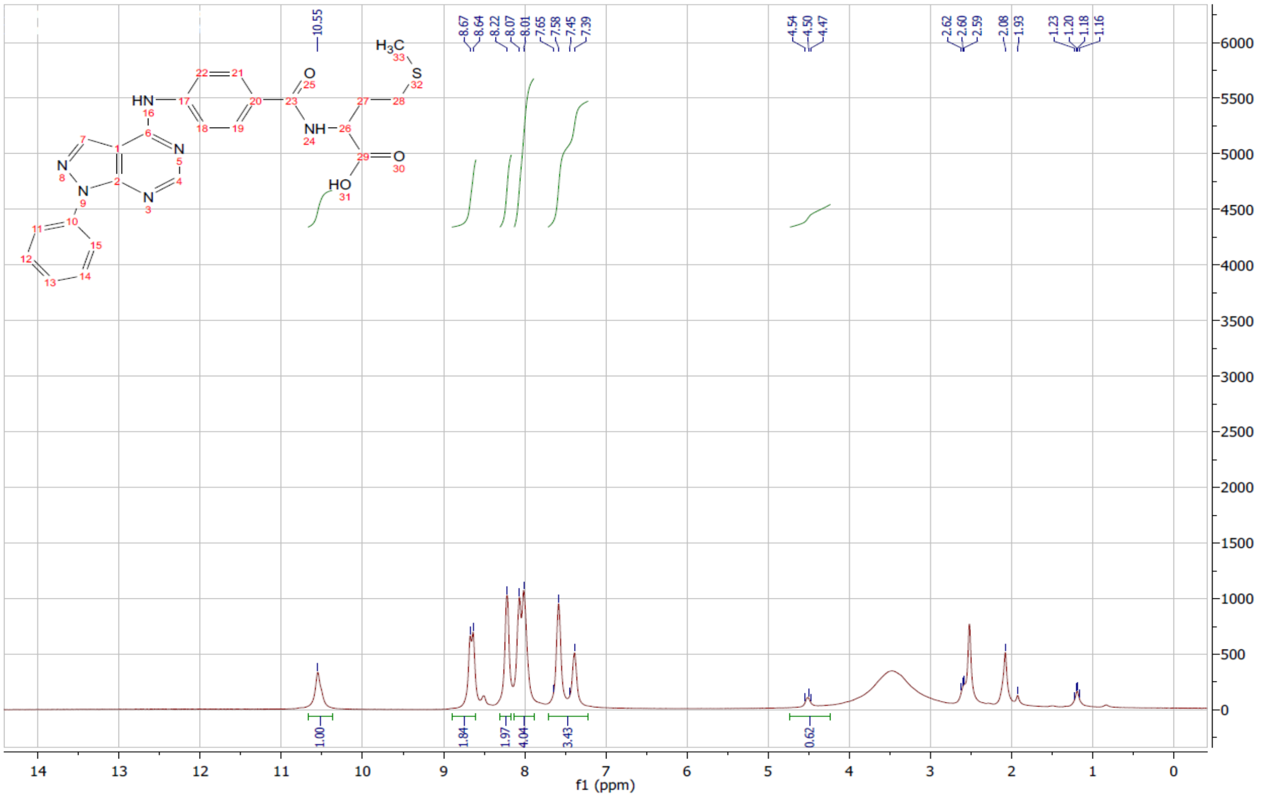

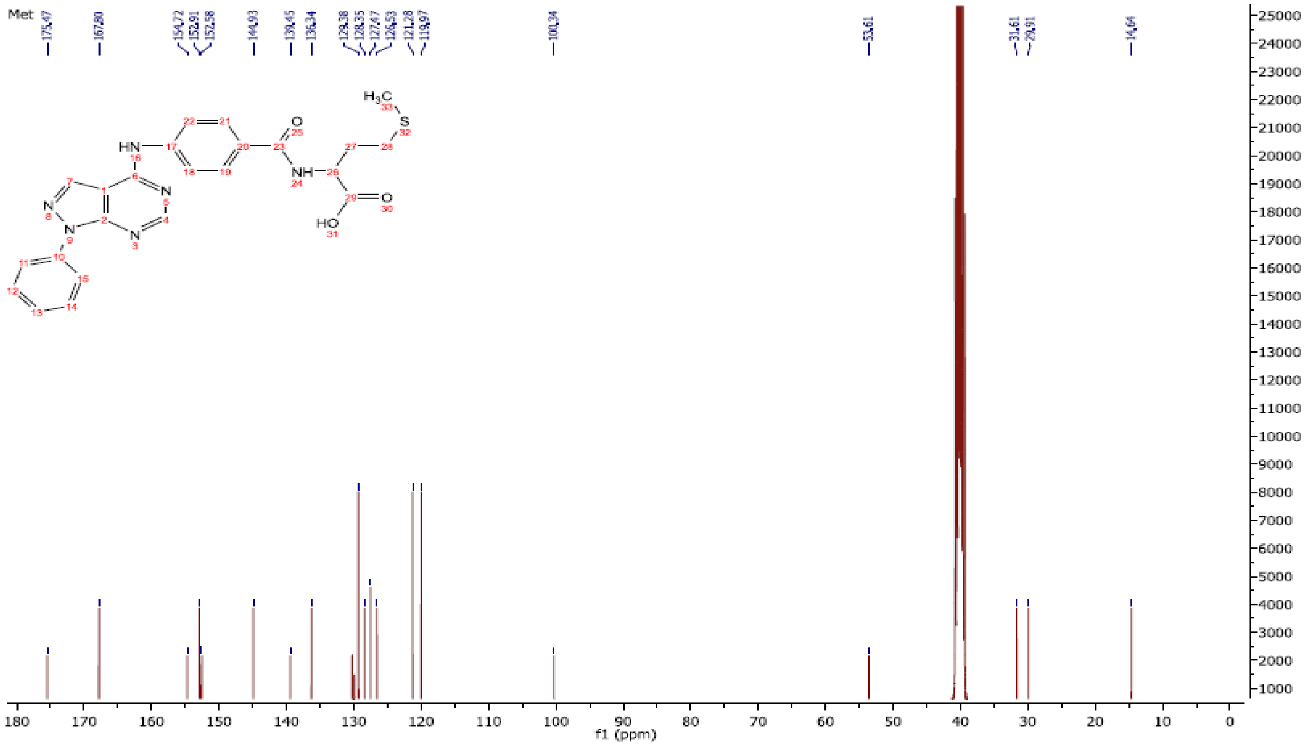

^{1}H NMR and ^{13}C NMR of compound (7_i)

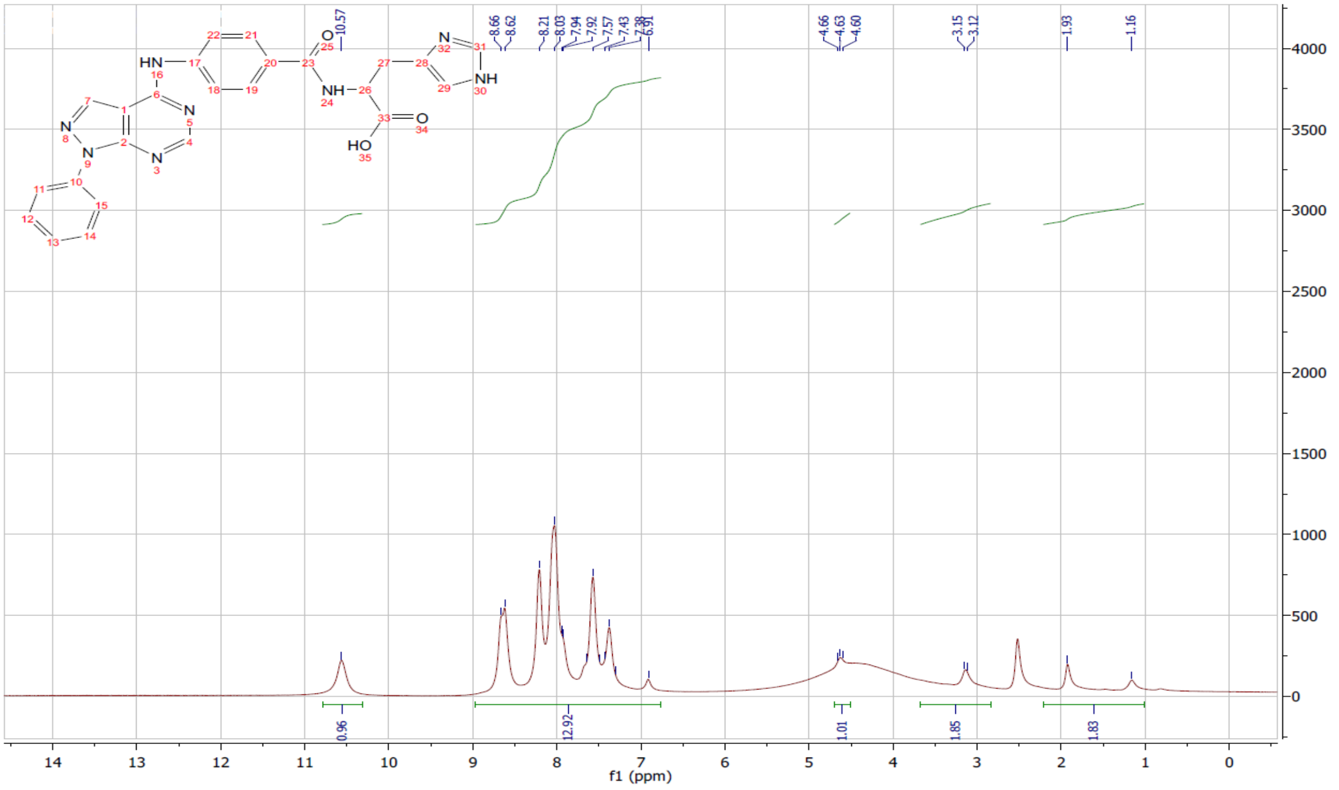

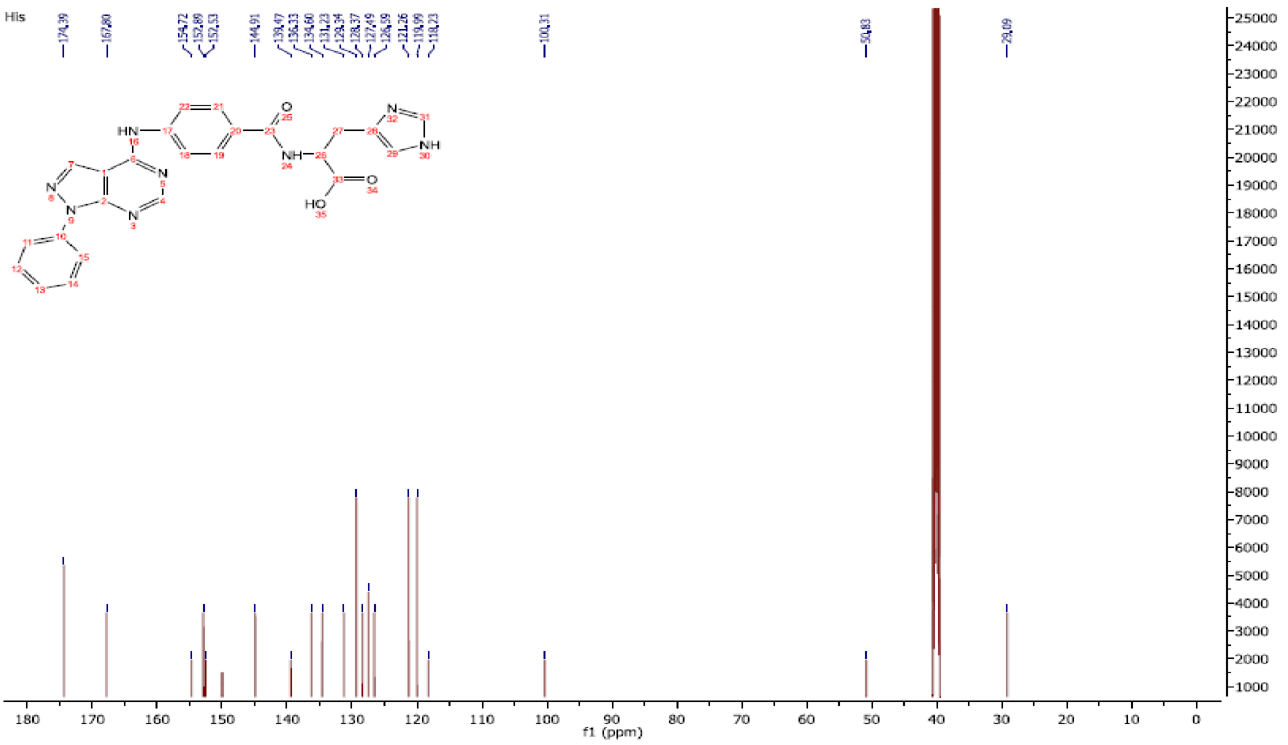

^{1}H NMR and ^{13}C NMR of compound (7_j)

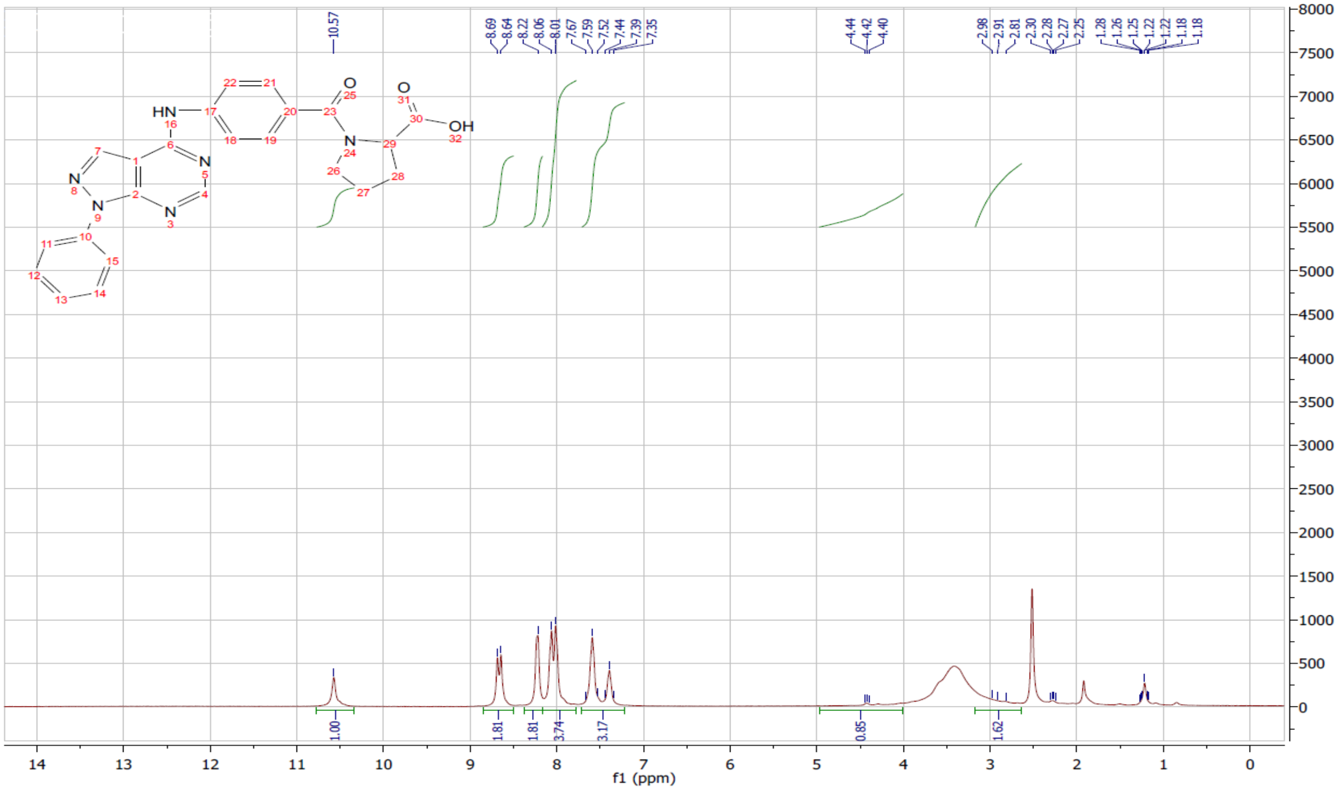

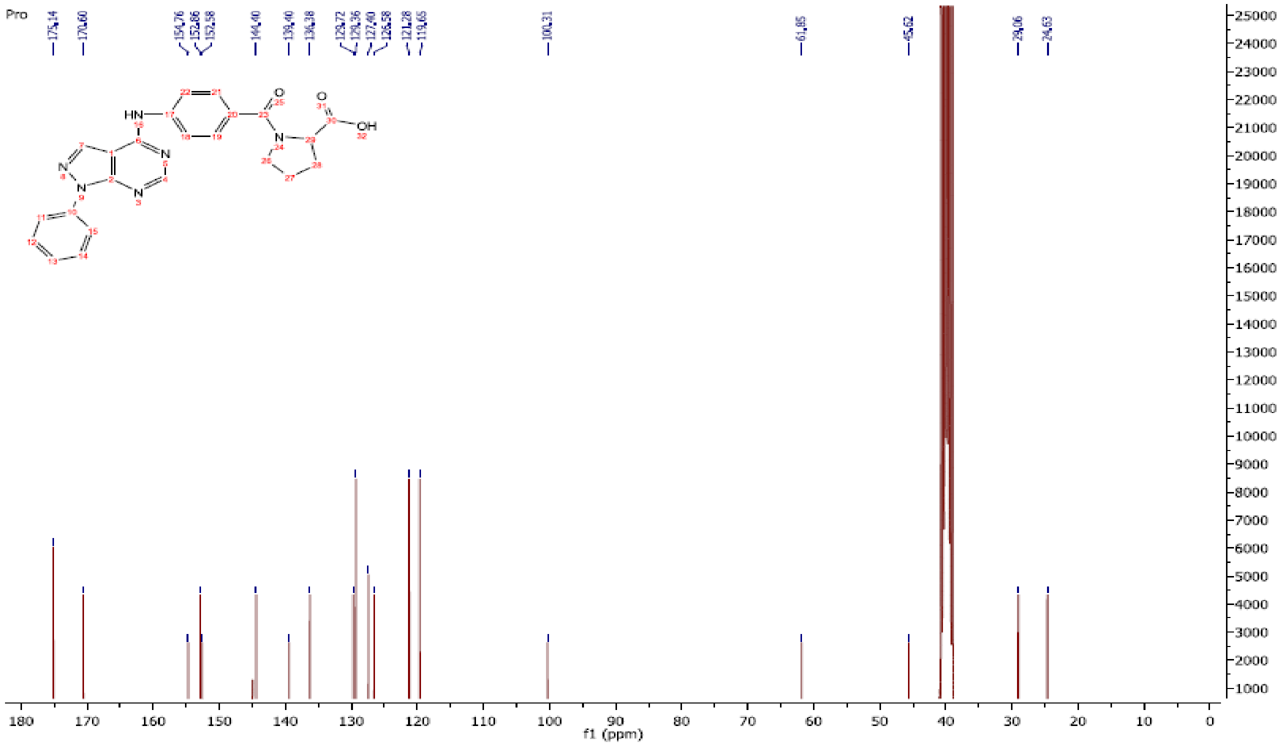

^{1}H NMR and ^{13}C NMR of compound (7_k)

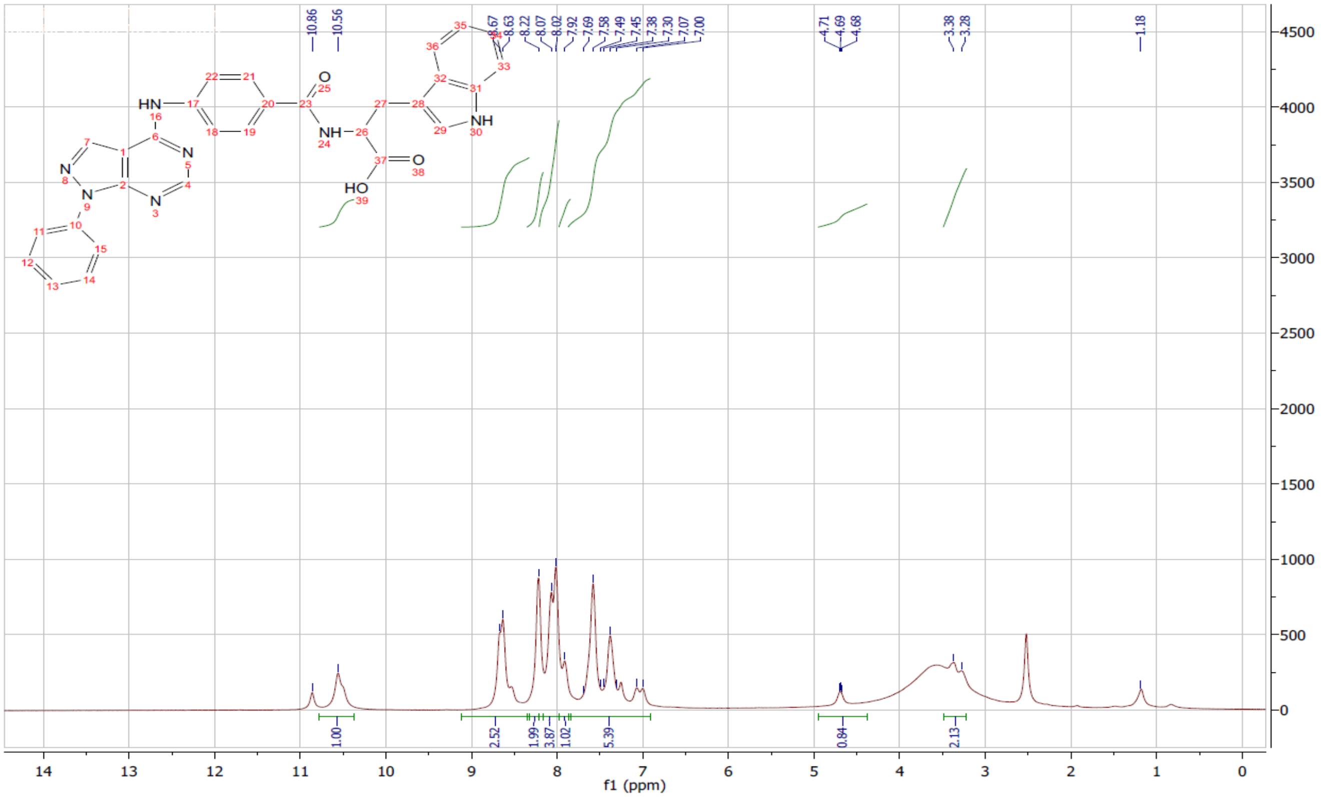

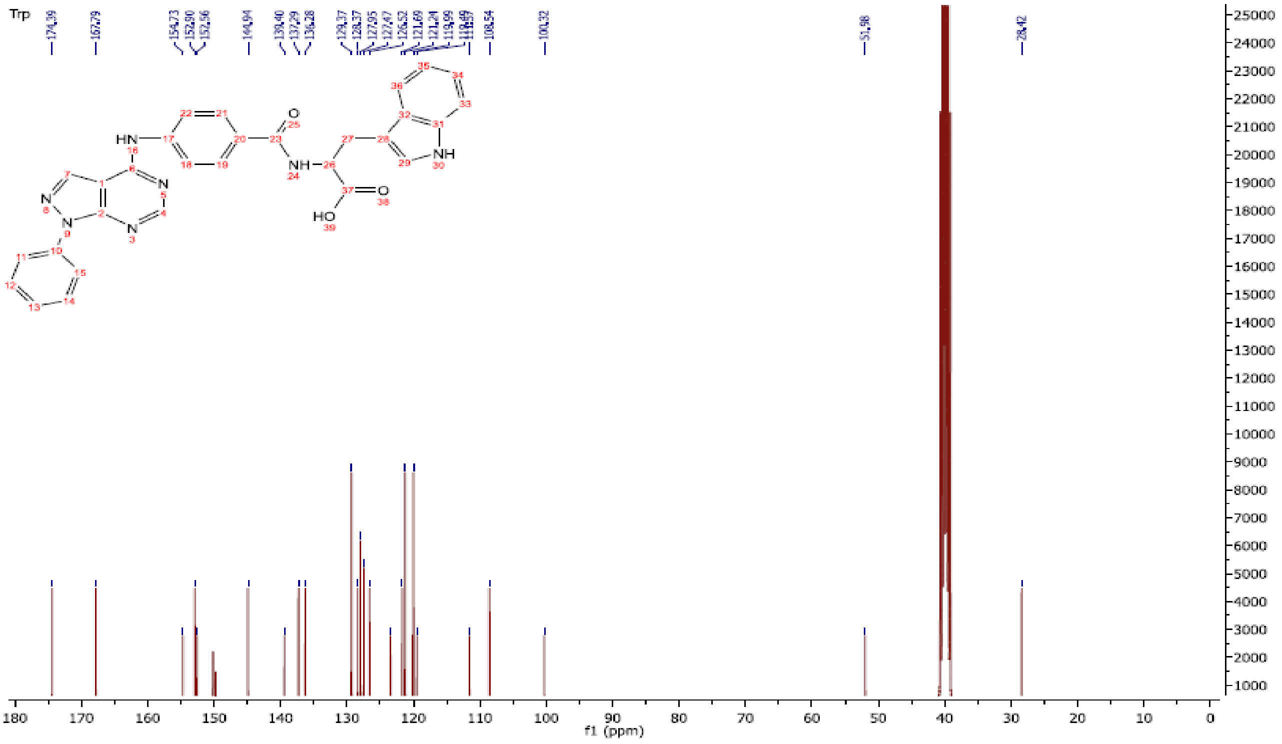

^{1}H NMR and ^{13}C NMR of compound (7l)

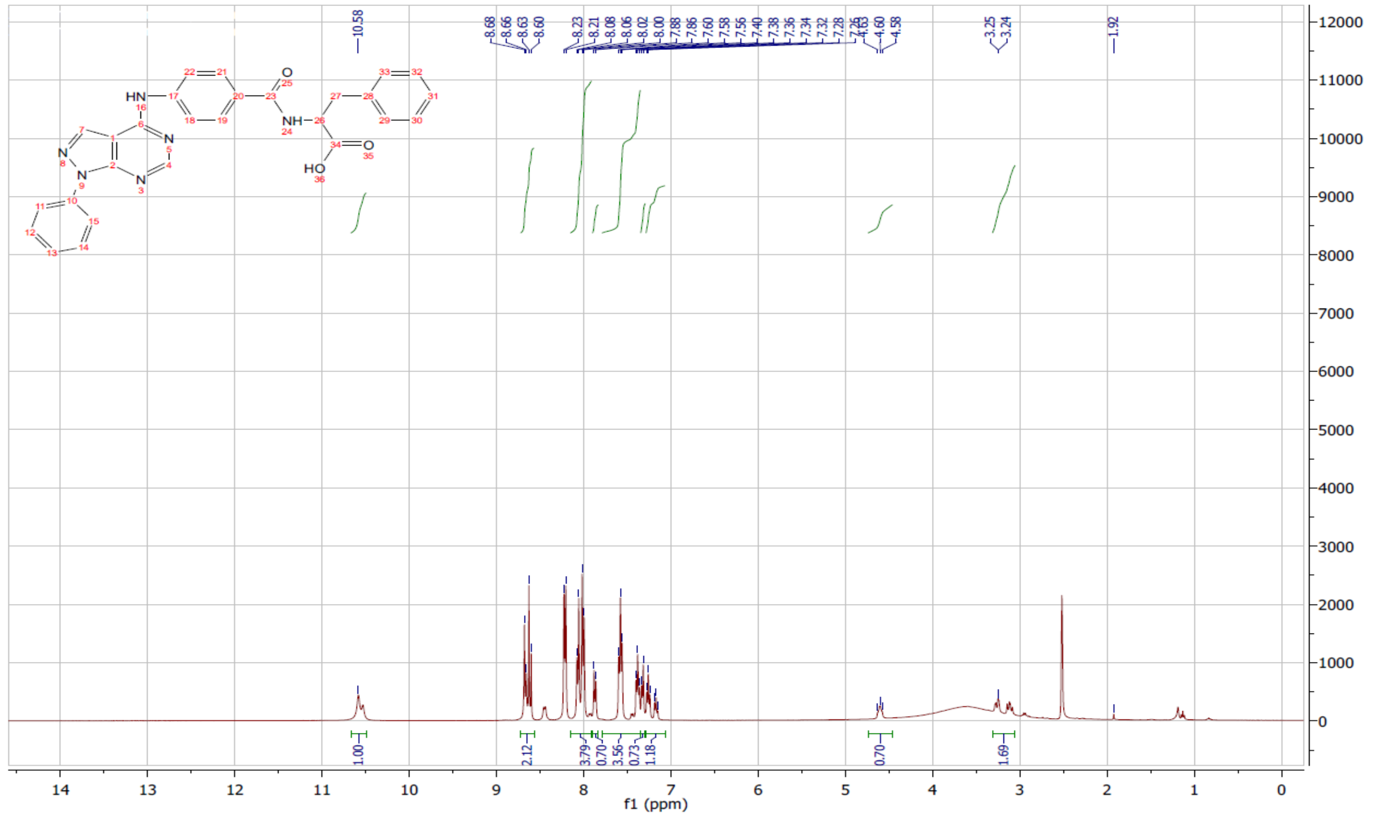

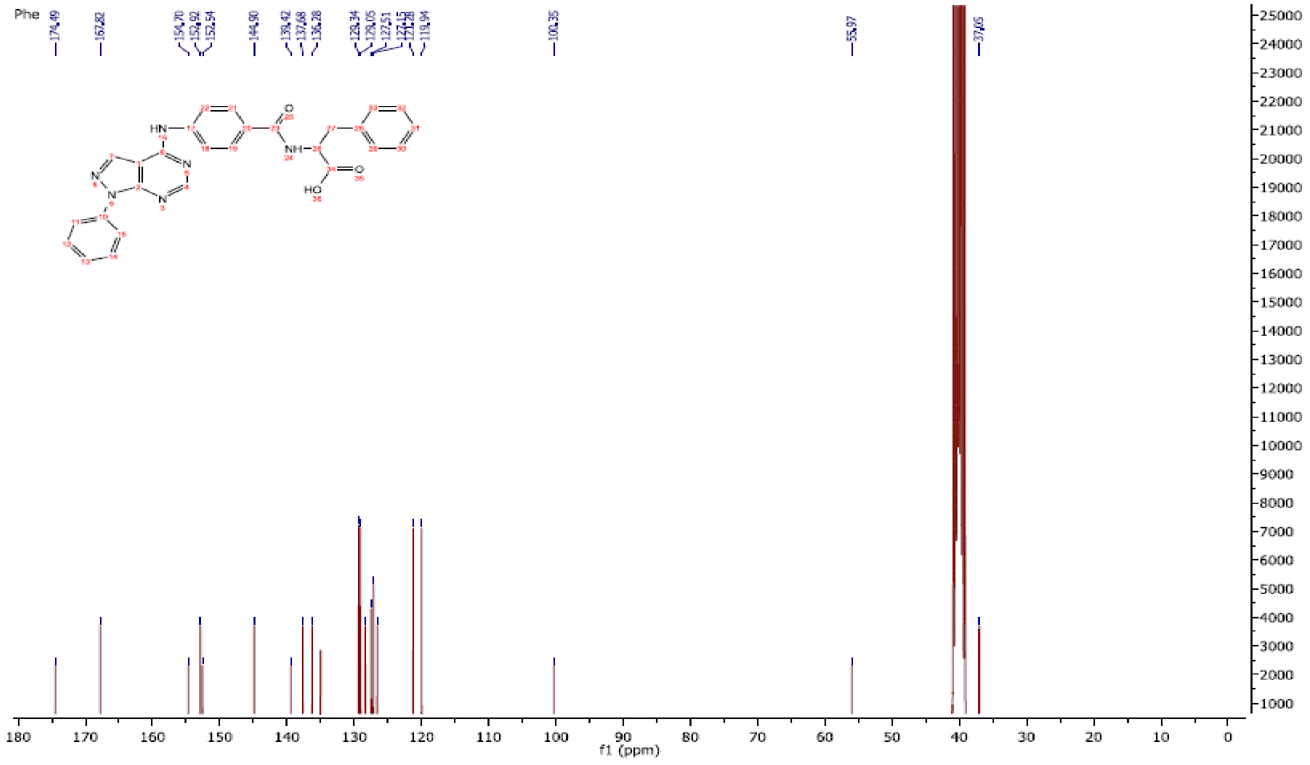

^{1}H NMR and ^{13}C NMR of compound (7_m)

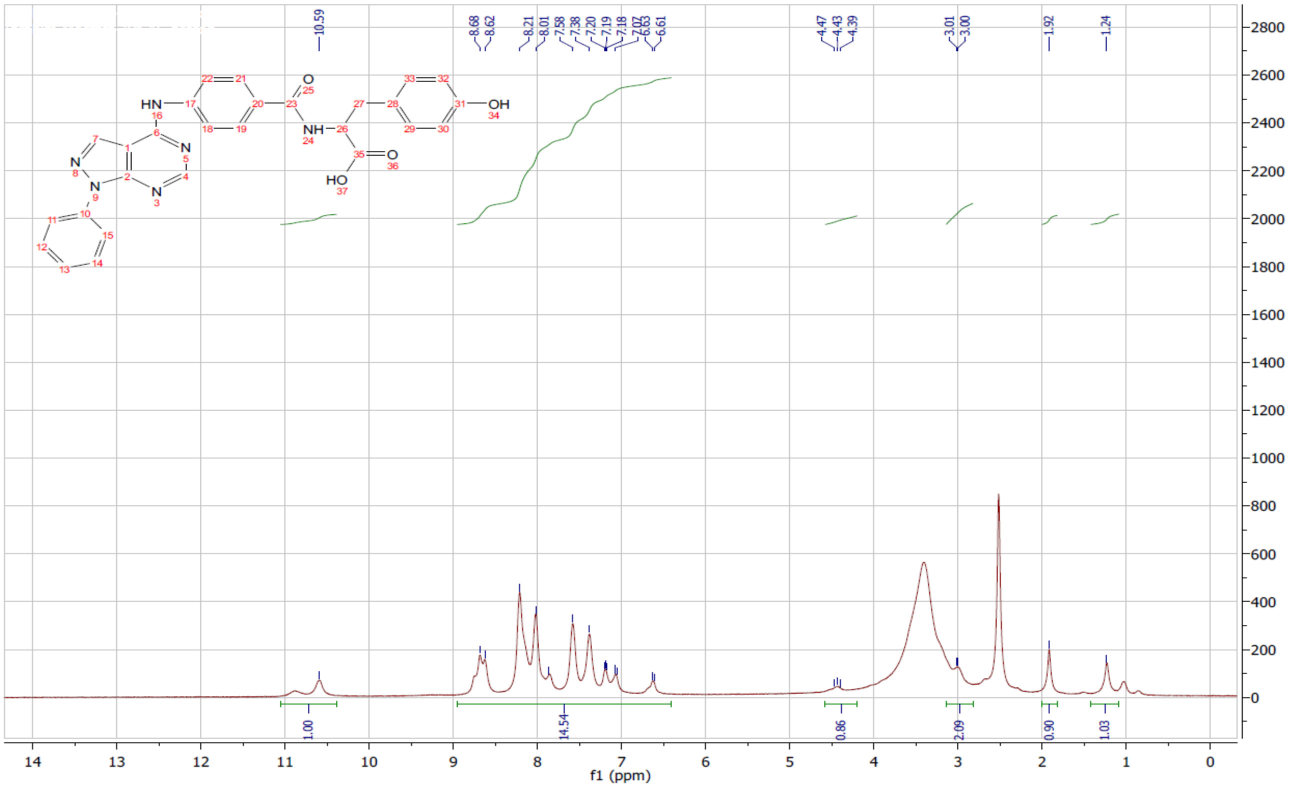

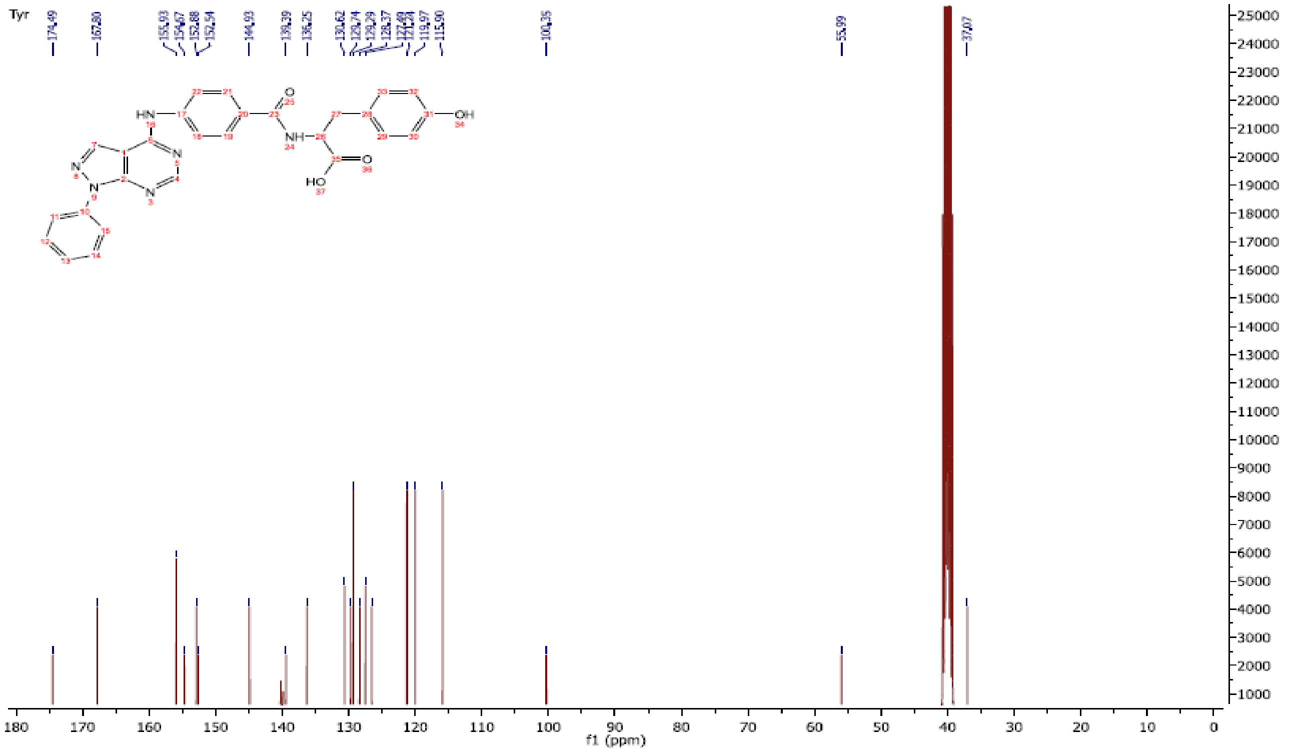

- Biological activity

DHFR [redacted]

code	IC50	conc	log	%inh	T2	T1	ΔT	RFU2	RFU1	ΔRFU	slope	K.Activity	EC
**6**		100	2	89	30	0	30	11.38	0	11.38	3.3333	13.6561	120
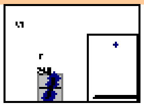													
		10	1	61	30	0	30	38.59	0	38.59	3.3333	46.3085	120
		1	0	29	30	0	30	71.26	0	71.26	3.3333	85.5129	120
		0.1	-1	17	30	0	30	82.57	0	82.57	3.3333	99.085	120
EC				0	30	0	30	100	0	100	3.3333	120	120

[redacted]

code	IC50	conc.uM	log conc	%inh	T2	T1	ΔT	RFU2	RFU1	ΔRFU	slope	K.Activity	EC
**7a**		100	2	81	30	0	30	19.02	0	19.02	3.3333	22.8242	120
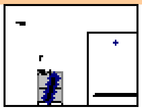													
		10	1	57	30	0	30	43.02	0	43.02	3.3333	51.6245	120
		1	0	30	30	0	30	69.51	0	69.51	3.3333	83.4128	120
		0.1	-1	12	30	0	30	88.32	0	88.32	3.3333	105.985	120
EC				0	30	0	30	100	0	100	3.3333	120	120

[redacted]

code	IC50	conc.uM	log conc	%inh	T2	T1	ΔT	RFU2	RFU1	ΔRFU	slope	K.Activity	EC
**7b**		100	2	72	30	0	30	27.51	0	27.51	3.3333	33.0123	120
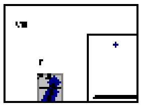													
		10	1	38	30	0	30	62.47	0	62.47	3.3333	74.9647	120
		1	0	20	30	0	30	79.58	0	79.58	3.3333	95.497	120
		0.1	-1	6.4	30	0	30	93.56	0	93.56	3.3333	112.273	120
EC				0	30	0	30	100	0	100	3.3333	120	120

[redacted]

code	IC50	conc.uM	log conc	%inh	T2	T1	ΔT	RFU2	RFU1	ΔRFU	slope	K.Activity	EC
**7c**		100	2	94	30	0	30	6.28	0	6.28	3.3333	7.53608	120

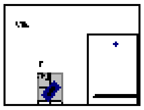

code	IC50	conc.uM	log conc	%inh	T2	T1	ΔT	RFU2	RFU1	ΔRFU	slope	K.Activity	EC
		10	1	80	30	0	30	19.71	0	19.71	3.3333	23.6522	120
		1	0	65	30	0	30	35.08	0	35.08	3.3333	42.0964	120
		0.1	-1	39	30	0	30	61.11	0	61.11	3.3333	73.3327	120
EC				0	30	0	30	100	0	100	3.3333	120	120

code	IC50	conc.uM	log conc	%inh	T2	T1	ΔT	RFU2	RFU1	ΔRFU	slope	K.Activity	EC
**7d**													
		100	2	95	30	0	30	4.63	0	4.63	3.3333	5.55606	120
		10	1	85	30	0	30	15.33	0	15.33	3.3333	18.3962	120
		1	0	66	30	0	30	33.71	0	33.71	3.3333	40.4524	120
		0.1	-1	42	30	0	30	58.42	0	58.42	3.3333	70.1047	120
EC				0	30	0	30	100	0	100	3.3333	120	120

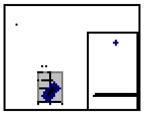

code	IC50	conc.uM	log conc	%inh	T2	T1	ΔT	RFU2	RFU1	ΔRFU	slope	K.Activity	EC
**7e**		100	2	89	30	0	30	11.04	0	11.04	3.3333	13.2481	120
		10	1	72	30	0	30	28.03	0	28.03	3.3333	33.6363	120
		1	0	57	30	0	30	43.26	0	43.26	3.3333	51.9125	120
		0.1	-1	28	30	0	30	72.06	0	72.06	3.3333	86.4729	120
EC				0	30	0	30	100	0	100	3.3333	120	120

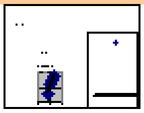

code	IC50	conc.uM	log conc	%inh	T2	T1	ΔT	RFU2	RFU1	ΔRFU	slope	K.Activity	EC
**7f**		100	2	94	30	0	30	6.06	0	6.06	3.3333	7.27207	120
		10	1	86	30	0	30	14.13	0	14.13	3.3333	16.9562	120
		1	0	66	30	0	30	34.28	0	34.28	3.3333	41.1364	120
		0.1	-1	44	30	0	30	55.76	0	55.76	3.3333	66.9127	120

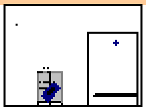

EC				0	30	0	30	100	0	100	3.3333	120	120

code	IC50	conc.uM	log conc	%inh	T2	T1	ΔT	RFU2	RFU1	ΔRFU	slope	K.Activity	EC
**7g**		100	2	84	30	0	30	15.75	0	15.75	3.3333	18.9002	120
		10	1	70	30	0	30	29.68	0	29.68	3.3333	35.6164	120
		1	0	55	30	0	30	44.64	0	44.64	3.3333	53.5685	120
		0.1	-1	37	30	0	30	62.85	0	62.85	3.3333	75.4208	120
EC				0	30	0	30	100	0	100	3.3333	120	120

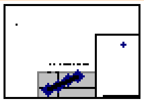

code	IC50	conc.uM	log conc	%inh	T2	T1	ΔT	RFU2	RFU1	ΔRFU	slope	K.Activity	EC
**7h**		100	2	81	30	0	30	19.17	0	19.17	3.3333	23.0042	120
		10	1	54	30	0	30	45.58	0	45.58	3.3333	54.6965	120
		1	0	36	30	0	30	63.67	0	63.67	3.3333	76.4048	120
		0.1	-1	21	30	0	30	78.93	0	78.93	3.3333	94.7169	120
EC				0	30	0	30	100	0	100	3.3333	120	120

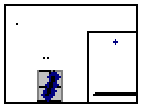

code	IC50	conc.uM	log conc	%inh	T2	T1	ΔT	RFU2	RFU1	ΔRFU	slope	K.Activity	EC
**7i**		100	2	94	30	0	30	5.99	0	5.99	3.3333	7.18807	120
		10	1	80	30	0	30	19.93	0	19.93	3.3333	23.9162	120
		1	0	63	30	0	30	37.21	0	37.21	3.3333	44.6524	120
		0.1	-1	35	30	0	30	65.31	0	65.31	3.3333	78.3728	120
EC				0	30	0	30	100	0	100	3.3333	120	120

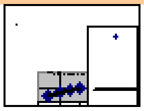

code	IC50	conc.uM	log conc	%inh	T2	T1	ΔT	RFU2	RFU1	ΔRFU	slope	K.Activity	EC
**7j**		100	2	93	30	0	30	7.27	0	7.27	3.3333	8.72409	120
		10	1	80	30	0	30	19.52	0	19.52	3.3333	23.4242	120

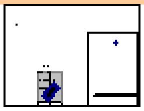

		1	0	64	30	0	30	35.81	0	35.81	3.3333	42.9724	120
		0.1	-1	41	30	0	30	59.28	0	59.28	3.3333	71.1367	120
EC				0	30	0	30	100	0	100	3.3333	120	120

code	IC50	conc.uM	log conc	%inh	T2	T1	ΔT	RFU2	RFU1	ΔRFU	slope	K.Activity	EC
**7k**		100	2	77	30	0	30	23.01	0	23.01	3.3333	27.6123	120
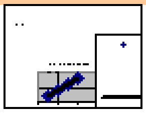		10	1	60	30	0	30	39.59	0	39.59	3.3333	47.5085	120
		1	0	37	30	0	30	62.51	0	62.51	3.3333	75.0128	120
		0.1	-1	21	30	0	30	79.43	0	79.43	3.3333	95.317	120
EC				0	30	0	30	100	0	100	3.3333	120	120

code	IC50	conc.uM	log conc	%inh	T2	T1	ΔT	RFU2	RFU1	ΔRFU	slope	K.Activity	EC
**7l**		100	2	92	30	0	30	8.41	0	8.41	3.3333	10.0921	120
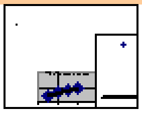		10	1	81	30	0	30	19.44	0	19.44	3.3333	23.3282	120
		1	0	68	30	0	30	31.85	0	31.85	3.3333	38.2204	120
		0.1	-1	34	30	0	30	66.03	0	66.03	3.3333	79.2368	120
EC				0	30	0	30	100	0	100	3.3333	120	120

code	IC50	conc.uM	log conc	%inh	T2	T1	ΔT	RFU2	RFU1	ΔRFU	slope	K.Activity	EC
**7m**		100	2	86	30	0	30	14.36	0	14.36	3.3333	17.2322	120
		10	1	73	30	0	30	27.16	0	27.16	3.3333	32.5923	120
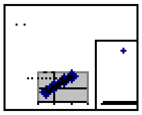		1	0	52	30	0	30	47.88	0	47.88	3.3333	57.4566	120
		0.1	-1	36	30	0	30	63.82	0	63.82	3.3333	76.5848	120
EC				0	30	0	30	100	0	100	3.3333	120	120

6	y = 24.624x + 36.737
7a	y = 23.439x + 33.312
7b	y = 21.526x + 23.456
7c	y = 17.986x + 60.462
7d	y = 17.975x + 62.99
7e	y = 19.829x + 51.488
7f	y = 16.925x + 63.98
7g	y = 15.626x + 53.957
7h	y = 19.737x + 38.293
7i	y = 19.524x + 58.128
7j	y = 17.232x + 60.914
7k	y = 19.218x + 39.255
7l	y = 18.527x + 59.304
7m	y = 16.91x + 53.24 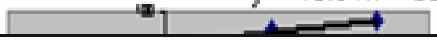
MTX	y = 15.083x + 71.386 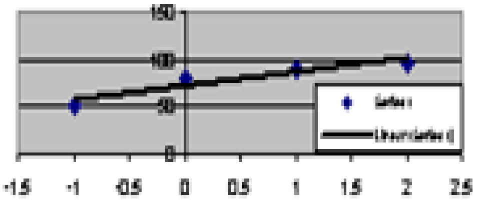

Lab Report

s	Compound			western blotting				β-actin
	code	Cells	conc	OD				
				DHFR	Casp3	Bax	Bcl2	
1	VIIf/MCF7			0.346	0.575	0.346	0.501	
2	Xe/MCF7			0.492	0.641	0.289	0.274	
3	Cont.MCF7			0.808	0.171	0.152	0.762	

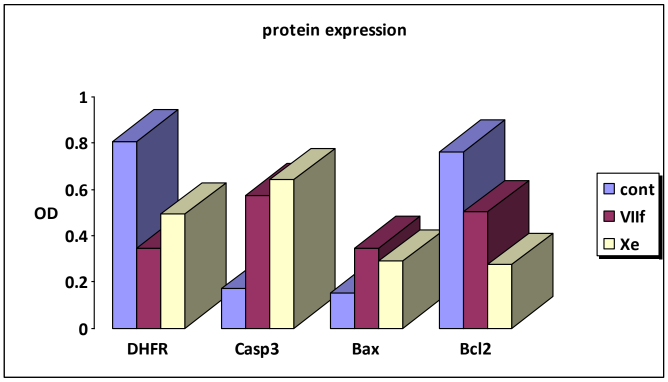

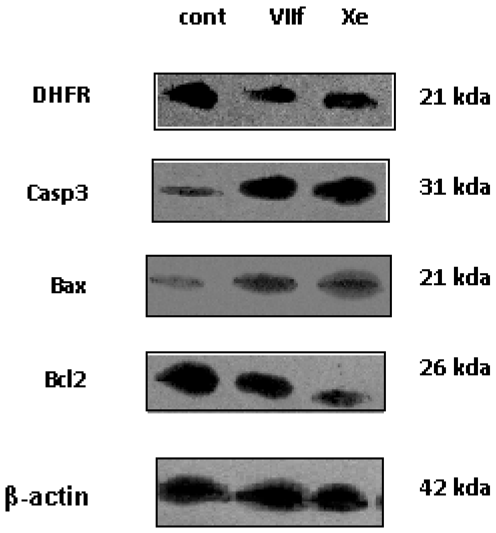

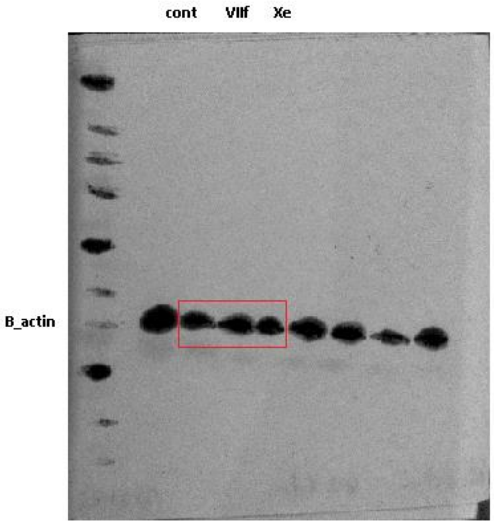
cont
VIIf
Xe
B_actin

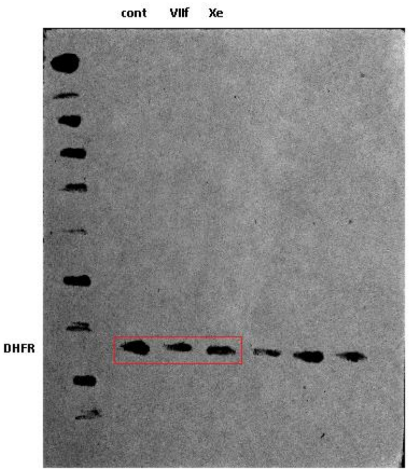
cont
VIIf
Xe
DHFR

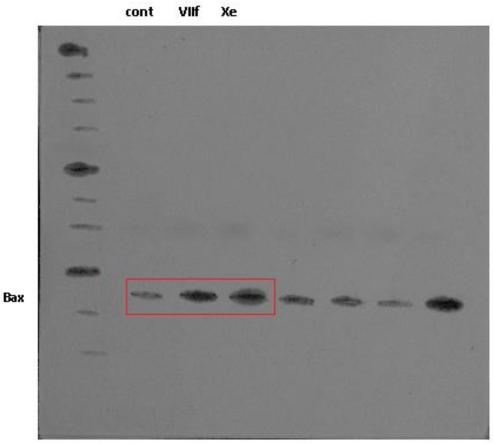
cont
VIIf
Xe
Bax

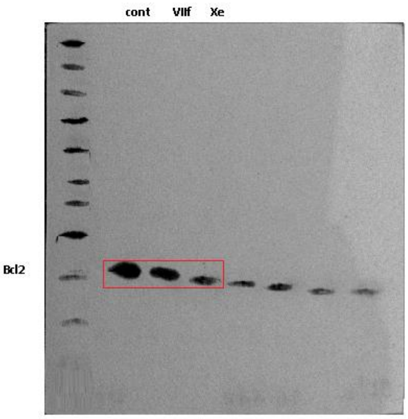
cont
VIIf
Xe
Bcl2

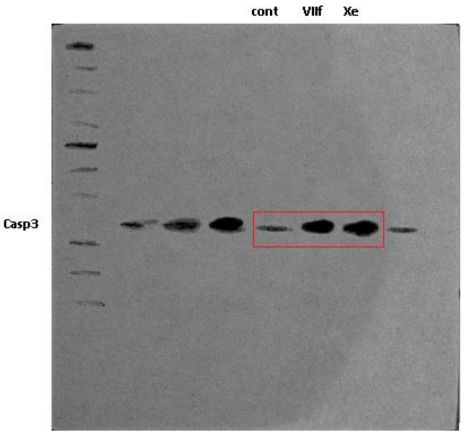
cont
VIIf
Xe
Casp3

Detailed Results:

Gel electrophoresis and immuno-blot analysis of proteins (Western Blot) (Burnette, 1981; Sambrook et al., 1989).

Principle:

Sodium dodecyl sulfate polyacrylamide gel electrophoresis (SDS-PAGE) is used to separate proteins based on their size. When coupled with western blotting (immunoblotting), both are typically used to determine the presence and/or relative abundance of a target protein in a sample containing a complex mixture of proteins. In this technique, total protein in each sample is loaded and electrophoretically separated by applying an electric current which allows the proteins to migrate through the gel matrix. In order for the proteins to migrate through the gel, they are first denatured and negatively charged by exposure to a detergent such as SDS. A molecular weight marker that produces bands of known size is used to help identifying proteins of interest. After the protein components have been sufficiently separated, they can be transferred to a polyvinylidene fluoride (PVDF) membrane by applying an electric current to the gel so that the proteins migrate out of the gel onto the membrane. For detection of a specific protein on the membrane, a primary antibody against that protein is added to form a protein-antibody complex followed by the addition of a secondary antibody that binds to the complex through its antibody side. The secondary antibody is typically linked to an enzyme that produces luminescence upon the reaction with its substrate. The amount of the luminescence, directly proportional to the amount of the protein that reacted with the antibody, is captured by Biorad Imager

Reagents preparation:

- Lysis buffer: 10mM Tris, 100mM NaCl, 25mM ethylenediaminetetra aceticacid (EDTA), 25mM Ethylene glycol bis(2-aminoethyl) tetraacetic acid (EGTA),0.1 Sodium dodecyl sulfate (SDS), % 1% (v/v) Triton X-100, 2% (v/v) NP-40 (pH 7.4), with 1:200 protease inhibitor cocktail (Sigma) and 1:300 phosphatase inhibitor cocktail Tablet (Roche).
- Polyacrylamide gel prepared according to table (??)
- Protein marker (Thermo scientific)
- SDS Loading buffer (6X): 750 mM Tris-HCl (pH 6.8); 600 mM dithiothreitol (DTT); 12 % SDS; 0.012 % Bromophenol blue; 60 % glycerol
- Tris-Glycine SDS running buffer: 25 mM Tris; 192 mM glycine (electrophoresis grade) (pH 8.3 -8.4); 0.1% SDS.
- Tris-Glycine transfer buffer: 25 mM Tris; 192 mM glycine; 0.05% SDS; 15% methanol. Methanol was immediately added before the transfer.
- Tris Buffered Saline Tween (TBS-T): 10 mM Tris-HCl (pH 8.0); 150 mM NaCl; 0.1% Tween-20
- 5 % non-fat dry milk in TBS-T
- Primary antibodies for the proteins to be detected ....
- Secondary antibodies for the proteins to be detected
- ECL™ western blotting detection chemiluminescent substrate (PerkinElmer, USA).

Procedure:

- The experiment was terminated by lysing the cells in cold lysis buffer. The cells were then immediately frozen at −20 °C for 1 h for further lysis, and collected by cell scraper and sonicated 2×10s, followed by centrifugation at 4000 rpm for 10 min under cooling.

- Total protein concentrations were determined colorimetrically in the supernatant using Bradford method before proceeding to the western blotting.
- **Western blotting**
- Equal amounts (20 µg) of protein samples were mixed and boiled with SDS Loading buffer for 10 min, allowed to cool on ice and then loaded into SDS-polyacrylamide gel and separated by Cleaver electrophoresis unit (Cleaver, UK), transferred onto polyvinylidene fluoride (PVDF) membranes (BioRad) for 30 min using a Semi-dry Electroblotter (Biorad, USA) at 2.5 A and 25 V for 30 min.
- The membrane was blocked with 5% nonfat dry milk in TBS-T for two hours at RT, in order to reduce non-specific protein interactions between the membrane and the antibody.
- The membrane was incubated overnight at 4°C with primary antibodies (Cell Signaling Technology) and β-actin (Sigma). The blots were then washed for three times (10 min each) with TBS-T.
- The membrane was then incubated with the corresponding horse radish peroxidase (HRP)- linked secondary antibodies (Dako) for another hour at room temperature, followed by washing for three times (10 min each) with TBS-T
- The chemiluminescent Western ECL substrate (Perkin Elmer, Waltham, MA) was applied to the blot according to the manufacturer's recommendation. Briefly, the membranes were incubated for 1 min with a mixture of equal volumes from ECL solution A and ECL solution B.

- The chemiluminescent signals were captured using a CCD camera-based imager (Chemi Doc imager, Biorad, USA), and the bands intensities were then measured by ImageLab (Biorad)
- Protein-sized markers were used in all gels to localize the gel transfer regions for specific proteins and determine the transfer efficiency.

Table (1): Solutions for preparing resolving and stacking gels for Tris-glycine SDS-Polyacrylamide gel electrophoresis

Stacking Gel	Resolving gel 12%	Components (Used as ml)
3.4	3.3	Water
0.83	4.0	30% acrylamide
---	2.5	1.5 M Tris (pH 8.8)
0.63	---	0.5 M Tris (pH 6.8)
0.05	0.1	10% SDS
0.05	0.1	10% Amm Persulfate
0.005	0.004	TEMED

References:

Burnette, WN (**1981**) **"Western blotting": electrophoretic transfer of proteins from sodium dodecyl sulfate--polyacrylamide gels to unmodified nitrocellulose and radiographic detection with antibody and radioiodinated protein A**. *Anal Biochem* 112, 195-203.

Sambrook J, Fritsch EF and Maniatis T (**1989**) **Molecular cloning: a laboratory manual**. Cold Spring Harbor Laboratory. Cold Spring Harbor, New York.
